# Electrochemotherapy with bleomycin, oxaliplatin, or cisplatin in mouse tumor models, from tumor ablation to *in situ* vaccination

**DOI:** 10.3389/fimmu.2025.1470432

**Published:** 2025-02-11

**Authors:** Katja Uršič Valentinuzzi, Urška Kamenšek, Simona Kranjc Brezar, Chloe Heranney, Tilen Komel, Simon Buček, Maja Čemažar, Gregor Serša

**Affiliations:** ^1^ Department of Experimental Oncology, Institute of Oncology Ljubljana, Ljubljana, Slovenia; ^2^ Biotechnical Faculty, University of Ljubljana, Ljubljana, Slovenia; ^3^ Biological Engineering Department, Polytech Clermont-Ferrand, Aubiere, France; ^4^ Faculty of Mathematics, Natural Sciences and Information Technologies, University of Primorska, Izola, Slovenia; ^5^ Department of Cytopathology, Institute of Oncology Ljubljana, Ljubljana, Slovenia; ^6^ Faculty of Medicine, University of Ljubljana, Ljubljana, Slovenia; ^7^ Faculty of Health Sciences, University of Primorska, Izola, Slovenia; ^8^ Faculty of Health Sciences, University of Ljubljana, Ljubljana, Slovenia

**Keywords:** ablative therapies, electrochemotherapy, *in situ* vaccination, abscopal effect, electroporation, chemotherapeutic drugs, mouse tumor models, cell death

## Abstract

**Introduction:**

In addition to its direct cytotoxic effects, ablative therapies as electrochemotherapy (ECT) can elicit indirect antitumor effects by triggering immune system responses. Here, we comprehensively analyzed this dual effectiveness of intratumoral ECT with chemotherapeutic drugs bleomycin (BLM), oxaliplatin (OXA), and cisplatin (CDDP). Our aim was to determine if ECT can act as *in situ* vaccination and thereby induce an abscopal effect. By evaluating ECT’s potential for *in situ* vaccination, our goal was to pave the way for future advancements for its combination with emerging (immuno)therapies, leading to enhanced responses and outcomes.

**Methods:**

We employed two mouse tumor models, the immunologically cold B16F10 melanoma and 4T1 mammary carcinoma, to explore both local and systemic (i.e., abscopal) antitumor effects following equieffective intratumoral ECT with BLM, OXA, and CDDP. Through histological analyses and the use of immunodeficient and metastatic (for abscopal effect) mouse models, we identified and compared both the cytotoxic and immunological components of ECT’s antitumor efficiency, such as immunologically recognizable cell deaths (immunogenic cell death and necrosis) and immune infiltrate (CD11^+^, CD4^+^, CD8^+^, GrB^+^).

**Results:**

Differences in immunological involvement after equieffective intratumoral ECT were highlighted by variable kinetics of immunologically recognizable cell deaths and immune infiltrate across the studied tumor models. Particularly, the 4T1 tumor model exhibited a more pronounced involvement of the immune component compared to the B16F10 tumor model. Variances in the antitumor (immune) response were also detected based on the chemotherapeutic drug used in ECT. Collectively, ECT demonstrated effectiveness in inducing *in situ* vaccination in both tumor models; however, an abscopal effect was observed in the 4T1 tumor model only.

**Conclusions:**

This is the first preclinical study systematically comparing the immune involvement in intratumoral ECT’s efficiency using three distinct chemotherapeutic drugs in mouse tumor models. The demonstrated variability in immune response to ECT across different tumor models and chemotherapeutic drugs provides a basis for future investigations aimed at enhancing the effectiveness of combined treatments.

## 1 Introduction

Local ablative therapies can effectively convert a tumor into an *in situ* personalized vaccine (1, 2). *In situ* vaccination seeks to amplify tumor immunogenicity, generate tumor infiltrating lymphocytes, and drive a systemic antitumor immune response, directed against untreated disseminated nodules, known as the abscopal effect (3). However, the latter, though discussed frequently, occurs rarely. *In situ* vaccination is mediated by T cells and there are two obstacles that prevent T cells to specifically recognize and eliminate tumor cells: first, the inadequate priming of antitumor T cells by specialized dendritic cells, and second, the incapacity of primed T cells to recognize and eliminate tumor cells (4–6). It has been proposed that electrochemotherapy (ECT) has the potential to trigger a robust *in situ* vaccination (7, 8). Nonetheless, a comprehensive examination of ECT involving various chemotherapeutic drugs has not been undertaken, either in preclinical or clinical settings.

Pulsed electric fields (PEF) elicit therapeutically beneficial effects on cancers, providing a valuable option for diverse patient populations. Among the various PEF therapies, such as irreversible electroporation, gene electrotransfer, tumor-treating fields, and calcium electroporation, ECT stands out as the most extensively investigated and applied, both preclinical and clinical contexts (9). In ECT, the combination of reversible electroporation (EP) and chemotherapeutic drugs facilitates the uptake of non-permeant or low-permeant agents such as cisplatin (CDDP) and bleomycin (BLM), into tumor cells, leading to increased cytotoxicity. ECT is mainly used in Europe as a local ablative treatment for cutaneous and subcutaneous malignancies (10) and for bone and intra-abdominal malignancies (11). Two standard operating procedures have been developed so far (12, 13), and the therapy has been incorporated into the European Standard Operating Procedures of ECT (ESOPE) study (10).

In addition to the direct cytotoxic effects of ECT on tumor cells, various other indirect mechanisms have been investigated, such as vascular (14–16) and immunological. The mode of cell death following ECT initiates a cascade of events that can either stimulate or dampen the antitumor response and ECT has been shown previously to cause immunologically relevant cell death (8, 17, 18). Specifically, in contrast to immunologically non-recognizable, silent or tolerogenic (19) apoptosis, immunologically recognizable necrosis and/or immunogenic cell death have the potential to induce an *in situ* vaccination effect. This phenomenon involves the release of antigens from ablated tumor tissue, coupled with other immunologically significant events in tumor cells and within the tumor microenvironment, collectively priming the immune system (5, 20). The priming exerts a favorable impact on both local and distant disease control. Despite successful immune system priming, descriptions of systemic effects following ECT are scarce in the literature (8, 21–23). Consequently, there has been a proposal and demonstration of the benefits of combining local ablative therapies like ECT with immunotherapies (24–26). Among them, immunostimulators such as cytokines IL-12 and IL-2 (27, 28) as well as check-point inhibitors (29, 30) have been employed in veterinary and human oncology.

In an ongoing effort to achieve optimal antitumor responses with minimal side effects, the range of drugs utilized in ECT has continuously expanded. In our previous studies, we introduced OXA as a third-generation platinum-based chemotherapeutic drug in intratumoral ECT, demonstrating comparable cytotoxic and immunomodulatory effects to CDDP in murine B16F10 melanoma (8, 31). Nevertheless, the underlying mechanisms of intratumoral ECT involving different chemotherapeutic drugs, particularly their impact on local and systemic antitumor effects, especially immune-mediated responses, remain unresolved.

**Figure 1:**
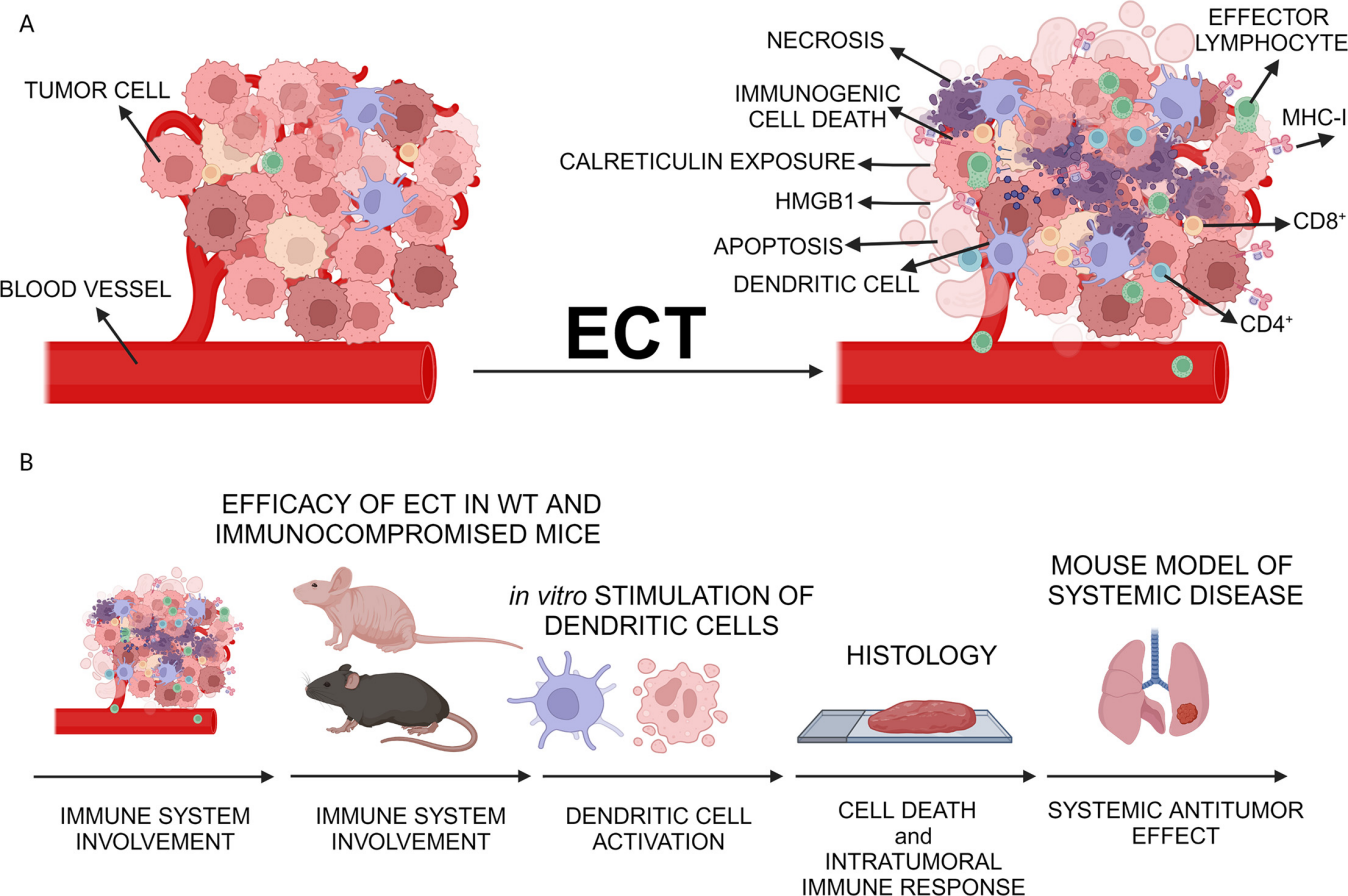
Study design. **(A)** Proposed local immune activation after ECT of tumors. **(B)** Steps to determine the ECT *in situ* vaccination potential. After determination of equieffective doses in ECT, local antitumor efficacy of ECT was assessed in both wild-type mice and immunocompromised mice. Dendritic cell stimulation was assessed *in vitro*, and histological analyses were performed to examine immune infiltrate post-ECT. Finally, the systemic antitumor efficacy was investigated in a murine model with lung metastases. Created with BioRender.com.

Therefore, our current study aims to comprehensively compare direct cytotoxic effects and, notably, immunologically significant events following intratumoral ECT with three distinct chemotherapeutic drugs – BLM, OXA and CDDP – utilizing two immunologically cold tumor models, B16F10 melanoma and 4T1 mammary carcinoma (8, 32–34). A prerequisite step for determining ECT’s *in situ* vaccination potential, was the establishment of equieffective *in vivo* doses for each chemotherapeutic. Subsequently the study was divided into four distinct steps (**Figure 1**): *in vitro* stimulation of dendritic cells, comparison of the ECT effectiveness in immunocompromised and immunocompetent mice, histological analyses at different time points post-ECT, and determination of systemic antitumor effectiveness in metastatic tumor models. By deciphering the time course and characteristics of the tumor response to intratumoral ECT, our ultimate goal was to strategize and implement combined treatments more effectively in the future.

## 2 Methods

### 2.1 Cell lines

All cell lines were purchased from American Type Culture Collection (ATCC, Manassas, VA, USA). B16F10 murine melanoma cells (CRL-6475; obtained in 2020; ATCC) were cultured in Advanced Dulbecco’s Modified Eagle Medium (DMEM; Gibco, Thermo Fisher Scientific, Waltham, MA, USA) and 4T1 mammary carcinoma cells (ATCC, CRL-2539; obtained in 2017) in Advanced Roswell Park Memorial Institute (RPMI) 1640 Medium (Gibco). Both media were supplemented with 5% fetal bovine serum (Thermo Fisher Scientific), 10 mM L-glutamine (GlutaMAX, Gibco), 100 U/mL penicillin (Grünenthal, Aachen, Germany), and penicillin-streptomycin (100×, Sigma-Aldrich, Merck, Darmstadt, Germany). Dendritic cells JAWS II (ATCC, CRL-11904; a kind gift of Angela Sousa, authenticated in 2023, IDEXX BioAnalytics, Westbrook, ME, USA) were cultured in alpha minimum essential medium with ribonucleosides, deoxyribonucleosides (Gibco), 4 mM L-glutamine (GlutaMAX, Gibco), 1 mM sodium pyruvate (Thermo Fisher Scientific) and 5 ng/ml murine GM-CSF (Miltenyi Biotec, Bergisch Gladbach, Germany) and 20% fetal bovine serum (Thermo Fisher Scientific). Cells were maintained in a 5% CO_2_ humidified incubator at 37°C. All cells were mycoplasma negative (MycoAlert™ PLUS Mycoplasma Detection Kit, Lonza, Basel, Switzerland).

### 2.2 Mouse models

Six- to seven-week-old (20–22 g) female C57BL/6NCrl, BALB/cAnNCrl and athymic nude mice (Crl: NU(NCr)-Foxn1nu) (Charles River Laboratories Italy S.R.L., Calco (Lecco), Italy) were used. All procedures were performed in compliance with 3Rs principle (reduce, replace, refine), guidelines for animal experiments of the EU Directives, the PREPARE guidelines and ARRIVE guidelines and the permission from the Administration of the Republic of Slovenia for Food Safety, Veterinary Sector and Plant Protection of the Ministry of Agriculture Forestry and Food of the Republic of Slovenia (Permissions No. U34401-35/2020/8 and U34401–3/2022/11) following the approval by the National ethical committee for experiments on animals. Mice were kept in individually ventilated cages at room temperature with a 12-h light–dark cycle in a specific pathogen-free environment with food and water *ad libitum*.

### 2.3 Establishment of B16F10 mCherry stable cell line

The B16F10 mCherry cell line was prepared using third-generation lentiviral plasmids: the transfer plasmid pLVX-EF1α-mCherry-N1 (Clontech, Takara, Kusatsu, Japan) encoding the mCherry fluorescent protein (mCherry) and the puromycin resistance gene for the selection of transduced cells, packaging plasmids pMDLg/pRRE and pMD2.G and the envelope plasmid pRSV-Rev. Lentiviral plasmids were propagated in *E. coli* under ampicillin selection and purified using the GeneJET Plasmid MiniPrep Kit (Thermo Fisher Scientific, Waltham, Massachusetts, USA).

The 293T cells (ATCC, CRL-3216) were used to generate viral particles. The cells were grown in complete DMEM (Gibco) to 80% confluence under standard conditions and transfected with plasmids using Lipofectamine 2000 transfection reagent (Invitrogen, Thermo Fisher Scientific, Waltham, MA, USA). A mixture (2 mL) of plasmids (15 µg pLVX-EF1α-mCherry-N1, 2.4 µg pMD2.G, 4 µg pMDLg/pRRE and 1.8 µg pRSV-Rev) and Lipofectamine 2000 in Opti-MEM (Gibco) was added dropwise to 293T cells according to the instructions provided with the transfection reagent. After 16 h the medium was replaced with 5 mL of complete DMEM. Medium containing viral particles was harvested 48 h and 72 h after transfection and replaced by 5 mL of DMEM. Thereafter, the media was centrifuged (500 × g for 10 min) and filtered through a 0.45 µm pore-size filter (Merck Millipore, Burlington, MA, USA) to remove cell debris. To concentrate lentiviral stocks, the Lenti-X™ Concentrator (Clontech Mountain View, CA, USA) was used and the concentration procedure was performed following manufacturer’s instructions. The samples were stored at -80°C.

For the transduction, B16F10 cells were grown in complete DMEM to 80% confluence at 6-well plate. On the day of transduction, the medium was replaced with the medium containing viral particles. After 24 h, the medium was replaced with a fresh medium. The next day, the expression of mCherry was detected by fluorescence microscopy (inverted fluorescence microscope IX70; Olympus, Tokyo, Japan) and 3 µg/mL of puromycin (Sigma-Aldrics) was added to the medium for the selection of the transduced cells. To prepare monoclonal culture, after 1 week of culturing under selective pressure, the cells were plated on a 96-well plate at a concentration of ~1 cell per well. Cells on the 96-well plate were cultured under the selective pressure of puromycin until colonies formed. Colonies were inspected using fluorescence microscopy and two clones with a high and uniform mCherry fluorescence intensity were selected for further propagation. mCherry-B16F10 cells were subcultured with selective pressure of 3 µg/mL of puromycin.

### 2.4 Electroporation *in vitro* in combination with chemotherapeutic drugs

EP *in vitro* on B16F10 mCherry cells was performed by delivering 8 100 µs long square wave pulses at 1300 V/cm and frequency of 1 Hz using an electric pulse generator (Jouan GHT beta, LEROY Biotech, Saint-Orens-de-Gameville, France) as described previously (18). Three different chemotherapeutic drugs were tested in combination with EP: bleomycin (BLM; Bleomycin sulfate, 10 mg, Selleckchem, Houston, TX, USA), cisplatin (CDDP; 1 mg/mL, Accord Healthcare Ltd., London, UK) and oxaliplatin (OXA; 5 mg/mL, Teva Pharmaceutical Industries Ltd., Jerusalem, Israel). Specifically, the inhibitory concentration for each drug that reduced cell survival to 50% (IC_50_) was adopted from our recent study (18).

### 2.5 *In vitro* stimulation of dendritic cells

Dendritic cells JAWS II were stained with 5-(and 6)-Carboxyfluorescein diacetate succinimidyl ester (CFSE) Kit (BioLegend, San Diego, CA, USA) according to manufacturers’s instructions and 4 × 10^5^ labeled dendritic cells JAWS II were seeded on T25 flasks. For stimulation, lipopolysaharide (LPS; 2 µg/ml, Merck, Darmstadt, Germany) was added to the medium. Four hours later, 2 × 10^5^ (control groups) or 4 × 10^5^ (ECT groups) B16F10 mCherry cells were added to dendritic cells. After 48 h, the media and cells were harvested by trypsinization, centrifuged (1400 rpm, 5 min) and washed twice with PBS. Additionally, cells were stained by Fixable Viability Dye eFluor™ 780 (Thermo Fisher Scientific) according to manufacturer’s instructions. The cells were analyzed using the BD FACSymphony™ A3 Cell Analyzer, and the data were analyzed using BD FACSDiva™ software, version 9.1 (both BD Biosciences, Franklin Lakes, NJ, USA). During the analysis, doublets and dead cells were excluded to ensure accurate results. Specifically, CFSE positive dendritic cells were analyzed for mCherry expression (CFSE^+^/mCherry^+^). The gating strategy is illustrated in **Supplementary Figure S1**. Fold change in the double positive cell population (mCherry^+^ and CFSE^+^) was calculated by dividing the percentage of the double positive cells in ECT groups with the percentage of double positive cells in control groups.

### 2.6 Tumor induction

Primary tumors were induced by subcutaneous injection of 0.5 × 10^6^ 4T1 or B16F10 cells in 100 µl of 0.9% NaCl saline. After formation of solid tumor, tumor volume was measured every 2-3 days using a Vernier caliper, and calculated using the following formula: a × b × c × π/6; where a, b and c represent three mutually orthogonal tumor diameters. For a mouse model of a systemic disease, seven days after the induction of primary B16F10 tumor, 3 × 10^5^ B16F10 cells (100 µl, 0.9% NaCl saline) were intravenously injected into the tail vein.

### 2.7 Electrochemotherapy *in vivo* and evaluation of antitumor effect

The treatment was performed when primary tumors reached 50-60 mm^3^. Mice were randomly divided into groups consisting of 6–8 animals, and were accommodated in individually ventilated cages. During treatment, mice were anesthetized with 1–3% isoflurane (Izofluran Torrex para 250 mL, Chiesi Slovenia, Ljubljana, Slovenia). The ECT consisted of intratumoral injection of the drug and application of electric pulses one minute later. A dose-finding study was conducted to determine equieffective suboptimal doses, i.e., doses that lead to a delay in tumor growth of ~20 days and do not cause complete tumor disappearance. Specifically, 40 µl of either the 0.9% NaCl saline (control group) or chemotherapeutic drugs BLM (Medac, Wedel, Germany; 0.4 µg, 0.75 µg, 1.5 µg, 2.5 µg, 5 µg, 7.5 µg), CDDP (2.5 µg, 5 µg, 10 µg, 30 µg, 40 µg, 50 µg) or OXA (10 µg, 20 µg, 30 µg, 60 µg, 85 µg, 100 µg) were tested. Electric pulses (2 sets of 4 square-wave pulses in perpendicular directions at a frequency of 1 Hz, a voltage-to-distance ratio of 1300 V/cm and a duration time of 100 μs) were delivered by ELECTRO Cell B10, Betatech electric pulse generator (Leroy Biotech, Orens-de-Gameville, France) using stainless steel plate electrodes with a 7-8 mm between the electrodes. A water-based ultrasound gel (ECO gel for ultrasound, Fiab, Florence, Italy) was used to ensure good conductivity at the contact of the electrodes with the skin overlaying the tumors. To determine the antitumor effect of ECT, tumor growth was followed until tumors reached 300 mm^3^ in volume. Animal weight and general health, which were determined through the examination of the coat and demeanor, were monitored daily. Humane endpoints were when the tumor reached 300 mm^3^, if a wet ulcer developed or if a mouse lost >15% weight of its initial weight. For each experiment, the specific number of animals is explicitly indicated in the graphs or/and figure captions.

For the evaluation of systemic antitumor effect, primary tumors were treated as described above. In B16F10 tumor model, metastatic model established through induced lung metastases was employed. In 4T1 model, spontaneous metastases were followed. In both models, when primary tumors reached approximately 300 mm^3^, surgical removal of primary treated tumors was performed. Mice were humanely euthanized, and their lungs were collected 20 days after therapy of the for B16F10 tumor model, or 40 days after therapy for 4T1 tumor model. After dissection, the lungs were rinsed in a Petri dish with physiological solution, dried with a clean paper towel, and placed in a container with Bouin’s solution. The presence of metastases on the lung surface was evaluated under stereomicroscope (specify the producer and the model). The number of lung metastases in each experimental group was divided by the number of lung metastases in the control group.

### 2.8 Histology

Mice were sacrificed, and primary tumors were excised at three different time points; 1, 3 and 7 days after the therapy. One-half of the collected tumor was used for immunofluorescence staining (IFC) and the other-half for hematoxylin and eosin (H&E) staining as well as immunohistochemical staining (IHC). For IFC, tumors were fixed in 4% paraformaldehyde (PFA; Alfa Aesar, Thermo Fisher Scientific) overnight, incubated in 30% sucrose for 24 h, embedded in Optimal cutting temperature compound (OCT compound, Sakura, Torrance, CA, USA) and snap frozen in dry ice. Consecutive, 14-μm thick tumor sections were cut using Leica CM1850 cryostat (Leica, Wetzlar, Germany). The consecutive sections were stained for blood vessel signature (CD31^+^), tumor infiltrating T cells (CD8^+^, CD4^+^) and immunogenic cell death [calreticulin (CLR^+^)]. For H&E and IHC staining, tumors were formalin-fixed and paraffin-embedded. The first section was used for the evaluation of necrosis through the tumor mass, and therefore, H&E staining was performed. The following sections were used for IHC to evaluate tumor infiltration of dendritic cells (CD11c^+^), granzyme positive effector immune cells as natural killer cells and cytotoxic T cells (GrB^+^) as well as evaluation of ICD marker, high-mobility group-box-1 protein (HMGB1) translocation. The used primary and secondary antibodies as well as antibody dilutions are listed in the **Supplementary Table S1**.

Specifically, for the IFC, the sections were firstly dried for 10 min at 37°C and washed twice for 5 min in 1 × PBS. Antigen retrieval was then performed by putting the slides in a hot citrate buffer (10 mM Sodium citrate in PBS, 0.5% Tween 20, pH 6, approx. 95°C) which was cooled down on air, at room temperature (RT) for 30 min followed by a 30 min cooling in RT water. After washing in PBS, the sections were blocked/permeabilized in blocking buffer (0.5% Triton X-100, 5% donkey serum, 22.52 mg/mL glycine in PBS) for 30 min at RT in a humidified chamber. Sections were blocked for 1 h at RT in blocking buffer (5% donkey serum, 22.52 mg/mL glycine in PBS) and then incubated overnight with primary antibodies (2% donkey serum, 22.52 mg/mL glycine in PBS) in a humidified chamber at 4°C. After washing three times in PBS, sections were incubated 1 h with secondary antibodies (2% donkey serum, 22.52 mg/mL glycine in PBS) at RT in a humidified chamber and then washed three times in PBS. Nuclei were counter-stained with Hoechst 33342 solution (Thermo Fisher Scientific) in PBS (3 µg/mL) for 10 min in the dark. After washing in PBS, slides were mounted with ProLong™ Glass Antifade Mountant (Thermo Fisher Scientific). For IFC staining of CLR, after the incubation with primary and secondary antibodies and washing in HBSS with calcium and magnesium (Thermo Fisher Scientific), the sections were counter-stained with Hoechst 33342 as well as wheat germ agglutinin conjugated with Alexa Fluor-488 (WGA in HBSS with calcium and magnesium; Invitrogen) for visualization of membranes. Samples (n = 3, 5 visual fields per sample) were imaged with LSM 800 confocal microscope (Carl Zeiss, Baden-Württemberg, Germany) with a 20 × objective (NA 0.8). Hoechst 33342, Alexa Fluor 488, Cy3 and Alexa Fluor 647 were excited with lasers with excitation wavelengths of 405 nm, 488 nm, 561 nm and 640 nm, respectively. To capture the emitted light Gallium Arsenide Phosphide (GaAsP) detector was used combined with a variable dichroic and filters at channel specific wavelengths: 410 – 545 nm (Hoechst 33342), 488 – 545 nm (Alexa Fluor 488), 565 – 620 nm (Cy3) and 645 – 700 nm (Alexa Fluor 647. The obtained images were visualized and analyzed with Imaris software (Bitplane, Belfast, UK). Cut-off values for each channel were determined based on negative control. The results are presented by fold-change (treatment group/control group).

For IHC, EXPOSE Rabbit-specific HRP/AEC or HRP/DAB detection IHC kit (Abcam, Cambridge, UK) was used. A brightfield microscope (BX-51 microscope, Olympus) connected to a DP72 CCD camera (Olympus) was used to capture images (n = 3, ≥5 visual fields per sample; 40 ×, 100 × and 400 × magnification). The necrotic area was analyzed blindly by three independent researchers, and the results are presented as the percent of necrosis, where the tumor area is annotated as 100%. To confirm the occurrence of immunogenic cell death, CLR and HMGB1 markers were not quantified; instead, their translocation was verified. The IHC/IFC positive cells were quantitatively evaluated by three independent researchers. The results are presented by fold-change (treatment group/control group).

### 2.9 Quantification of HMGB1 serum concentration

Quantification of HMGB1 serum concentrations was performed using an enzyme-linked immunosorbent assay (ELISA) kit (Chondrex, Woodinville, WA, USA) according to manufacturer’s recommendations. Specifically, blood samples (maximum volume of 300 ul) were taken 24 h and 72 h after the therapy via orbital sinus puncture and transferred by capillary tube to the serum-separating tubes (SST Microtainer^®^ blood collection tubes, BD biosciences, Franklin Lakes, NJ, USA). Serum was separated after 30 min incubation at RT by 10 min centrifugation at 1300× g and stored at –80°C until further analysis. Samples were diluted 1:1 and ran in technical replicates.

### 2.10 Statistical analysis

All graphical presentations and statistical analyses were made in GraphPad Prism 9 (GraphPad Software). All data were tested for distribution normality using the Shapiro-Wilk test. Data are presented as the arithmetic mean (AM) ± the standard error of the mean (SE). Comparison of means was performed with one-way ANOVA followed by Dunnett’s multiple comparisons test. Differences were considered significant at * p ≤ 0.05, ** p ≤ 0.01, *** p ≤ 0.001, **** p ≤ 0.0001. Sample size (n) represents biological replicates for each experiment and is presented in each figure legend. In Kaplan-Meier analysis (Survival Log-Rank Test), tumor volumes of 300 mm^3^ were counted as events for the construction of the curves. Additionally, tumor growth delay (time after treatment when tumors reached 200 mm^3^ in the treatment group/control group), was calculated. Two-sided Fisher’s exact test was used to compare proportions of metastasis-free mice in 4T1 spontaneous metastases model.

## 3 Results

### 3.1 Equieffective ECT with BLM, OXA and CDDP

**Figure 2:**
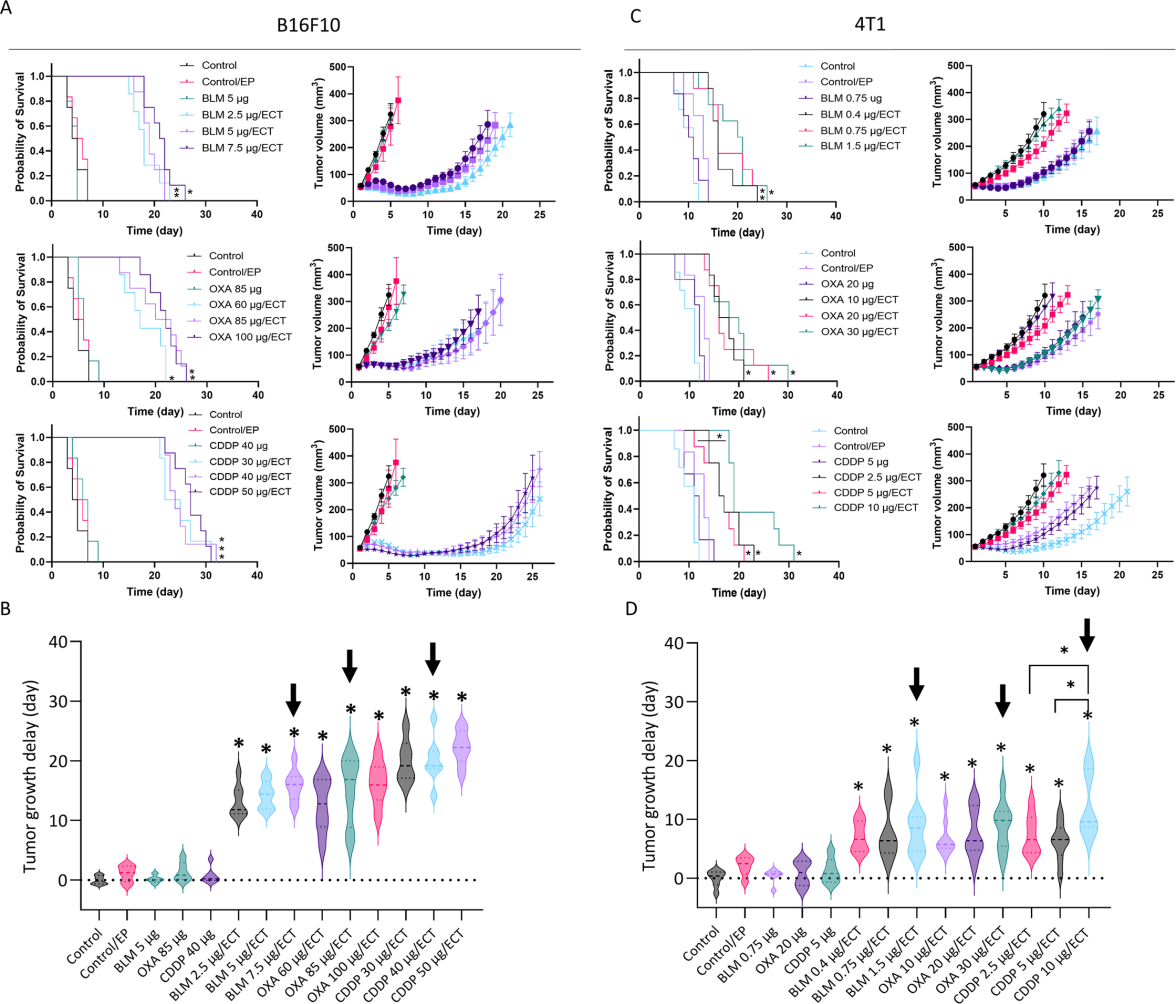
Determination of equieffective ECT doses of BLM, OXA and CDDP in B16F10 and 4T1 tumor models. Animal survival, growth curves and tumor growth delay are presented after ECT with three doses of the chemotherapeutic drugs in **(A, B)** B16F10 tumors and **(C, D)** 4T1 tumors. The survival of animals after ECT is presented with Kaplan-Meier graphs (n = 6-8; Survival Log-Rank Test; *p < 0.05, **p ≤ 0.01, ***p ≤ 0.001), tumor growth curves (n = 6-8; AM ± SE) and tumor growth delay with violin plots (n = 6-8; AM ± SE, *p < 0.05). Arrows indicate equieffective doses of ECT with BLM, OXA or CDDP that were used for the following experiments. BLM, bleomycin; OXA, oxaliplatin; CDDP, cisplatin.

To determine equieffective intratumoral ECT using suboptimal doses of BLM, OXA or CDDP in B16F10 and 4T1 tumors, three doses of each chemotherapeutic drug were tested. The doses were selected based on our preliminary data and published literature (8, 35). The selected equieffective doses of chemotherapeutic drugs that were used for subsequent experiments were 7.5 µg BLM, 85 µg OXA, or 40 µg CDDP for B16F10 tumors (**Figures 2A, B**, **Supplementary Figure S2A**, **Supplementary Table S2**). For 4T1 tumors, the selected doses were 1.5 µg BLM, 30 µg OXA, or 10 µg CDDP, demonstrating a lower drug dose requirement for the 4T1 tumor model compared to the B16F10 tumor model (**Figures 2C, D**, **Supplementary Figure S2B**, **Supplementary Table S2**). ECT with selected doses resulted in comparable tumor growth and survival in each tumor model. Moreover, survival of the ECT treated animals was comparable also between tested tumor models (**Figures 2A, C**). However, tumor growth delay was significantly higher in B16F10 tumor model compared to 4T1 model (**Figures 2B, D**).

### 3.2 ECT induces immunologically recognizable cell death

**Figure 3:**
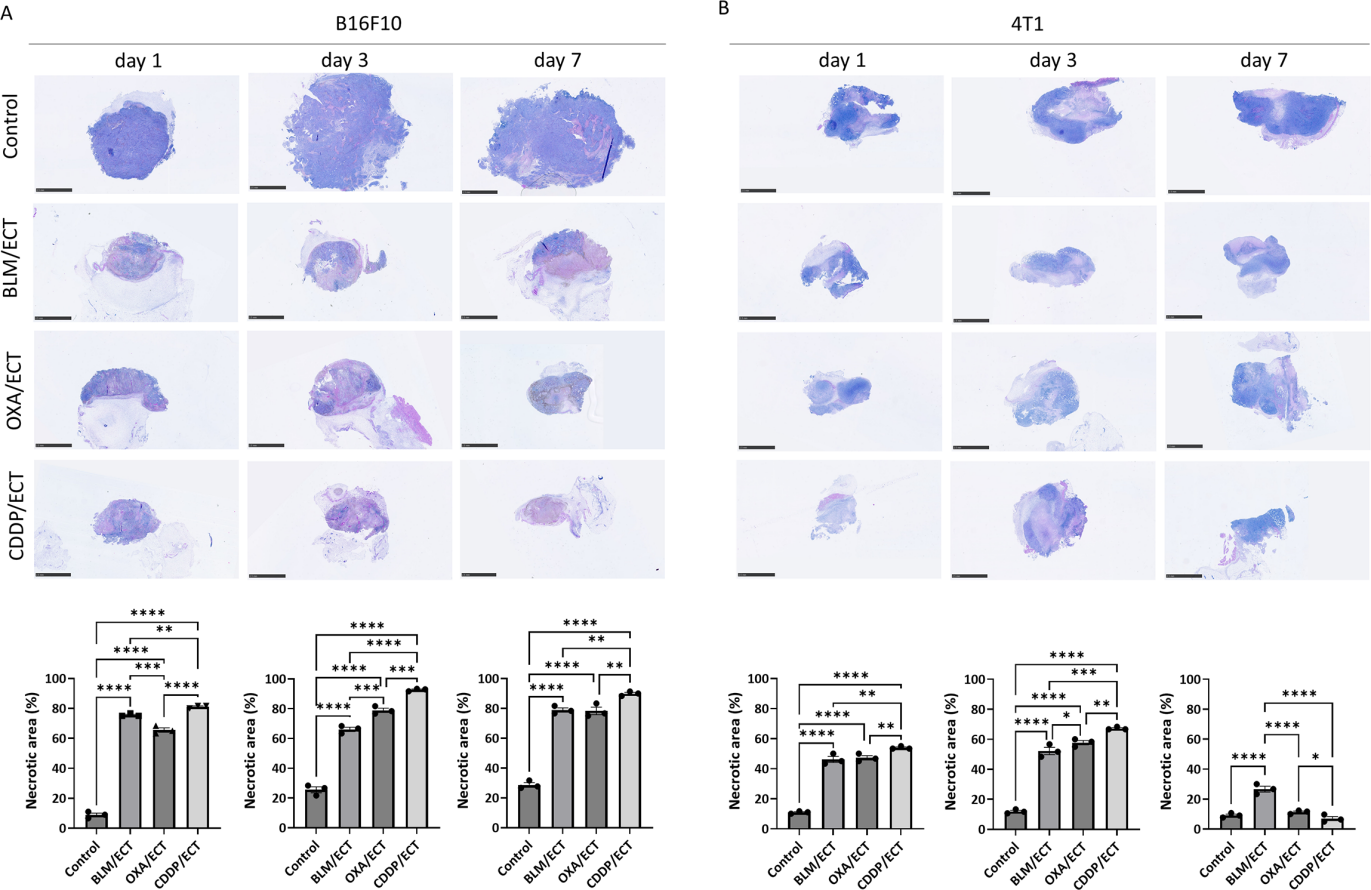
ECT induces necrosis. Necrotic areas one, three and seven days after ECT in **(A)** B16F10 and **(B)** 4T1 tumors. Scale bar: 2.5 mm. BLM, bleomycin; OXA, oxaliplatin; CDDP, cisplatin. (n = 3; AM ± SE and individual measurements are presented; *p ≤ 0.05, **p ≤ 0.01, ***p ≤ 0.001, ****p ≤ 0.0001).

Within the spectrum of immunogenic, tolerogenic, and silent cell deaths (19), our focus was particularly directed towards immunologically recognizable cell deaths, namely necrosis and immunogenic cell death. Initially, we evaluated the necrotic regions using H & E-stained sections one, three and seven days after the therapy (**Figures 3A, B**, **Supplementary Figures S3, B**). The application of ECT with BLM, OXA, or CDDP resulted in a significant increase in necrotic areas in both tumor models (**Figures 3A, B**). Approximately 70% of the B16F10 tumors were necrotic at all three time points after ECT, while the untreated control group showed only around 20% necrosis. In the 4T1 model more than 50% of the tumor mass was necrotic post-ECT at day one and three, whereas control tumors were 90% viable. Intratumoral administration of chemotherapeutic drugs without electric pulse application also resulted in increased necrotic areas at all three time points in both tumor models compared to the control untreated group, although to a lesser extent than ECT (**Supplementary Figures S3A, b**).

**Figure 4:**
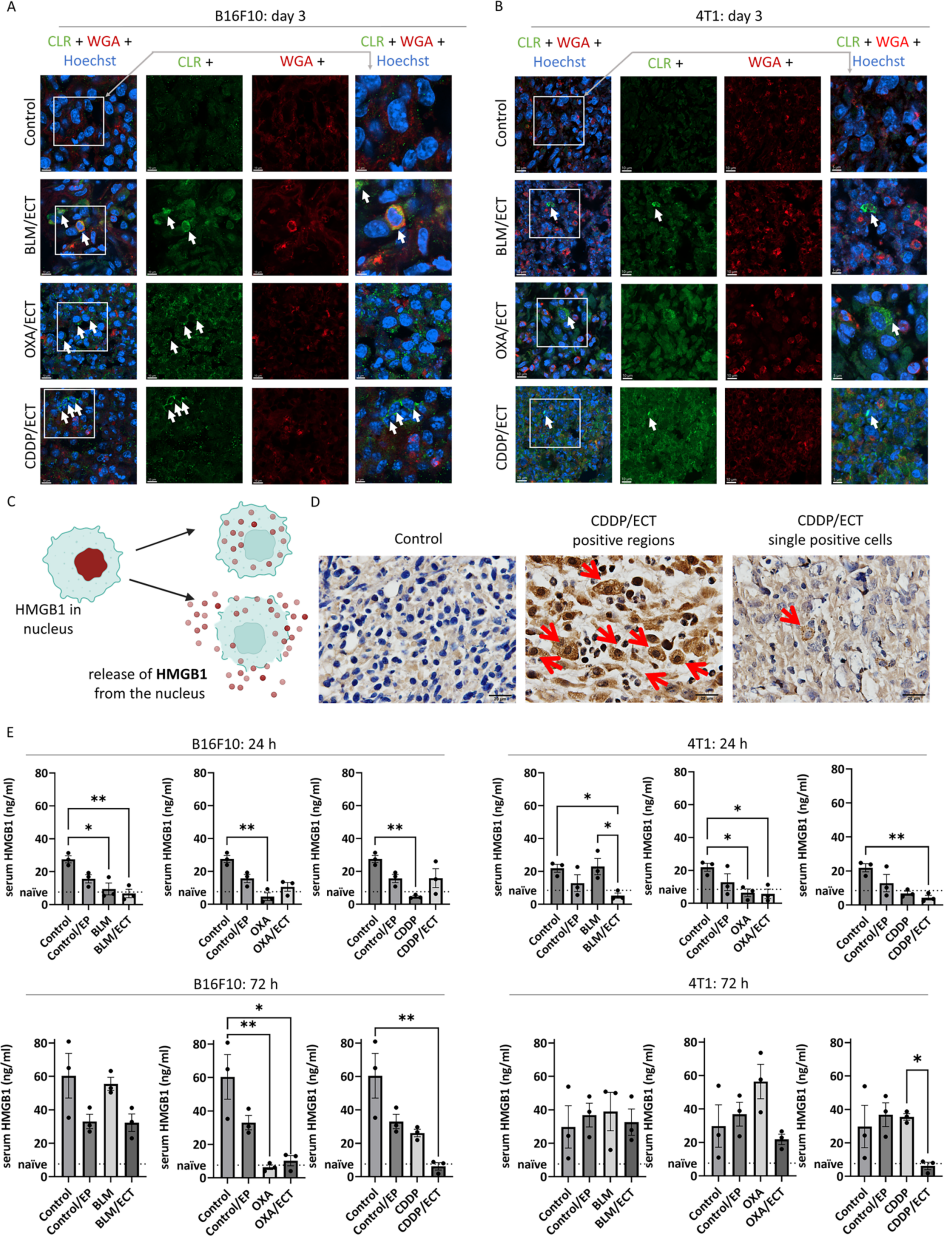
Immunogenic cell death is induced after ECT. Two markers of immunogenic cell death were investigated; calreticulin (CLR) exposure to plasma membrane and high-mobility group box 1 (HMGB1) protein release from dying cells. CLR was exposed to plasma membrane after ECT with BLM, OXA or CDDP in **(A)** B16F10 and **(B)** 4T1 tumors. Arrows indicate colocalization of CLR (green) and membranes (WGA, red). Scale bar: 10 µm (column 1-3) or 5 µm (column 4). **(C)** During immunogenic cell death, HMGB1 is released from nucleus as presented in the drawing. **(D)** Representative micrographies of HMGB1 positive 4T1 tumor regions as well as single tumors cells releasing HMGB1. Arrows indicate IHC positive signal. Scale bar: 20 µm. **(E)** Serum concentration of HMGB1 24 and 72 hours after the treatment in mice bearing B16F10 and 4T1 tumors. (n = 3; AM ± SE and individual measurements are presented *p ≤ 0.05, **p ≤ 0.01).

Subsequently, we exclusively assessed immunogenic cell death on day one and three post-ECT. Specifically, we investigated the translocation of CLR from the endoplasmic reticulum to the plasma membrane and the release of high-mobility group box 1 protein (HMGB1) from dying tumor cells. CLR translocation to the plasma membrane was observed in both tumor models following ECT, regardless of the specific chemotherapeutic drug employed (**Figures 4A, B**, **Supplementary Figures S4C, D**, **S5C, D**, **S6**). While CLR translocation was rarely observed after intratumoral application of OXA without electroporation, it was not detected after the intratumoral application of BLM or CDDP only (**Supplementary Figures S4A, B**, **S5A, B**, **S6**).

The second DAMP investigated in conjunction with immunogenic cell death was the release of HMGB1 from dying tumor cells, as depicted in **Figure 4C**. HMGB1 was exclusively detected in ECT-treated cells, exhibiting localization in the nuclear, cytosolic, and extracellular compartments (**Figure 4D**). Specifically, in the tumor cells of untreated tumors, we did not detect the HMGB1, whereas after ECT, we observed tumors regions with heightened HMGB1 levels as well as individual HMGB1 positive cells, indicative of immunogenic cell death. In addition to assessing HMGB1 in tumor cells, we also explored the presence of serum HMGB1 at 24 and 72 hours post-ECT as a potential systemic biomarker of immunogenic cell death (**Figure 4E**). While serum HMGB1 concentrations in naïve C57Bl/6 and Balb/c were 7.65 ng/mL and 8.51 ng/mL, respectively, the concentrations in untreated B16F10 and 4T1 tumor-bearing mice were approximately a threefold increased, which further escalated over time. Surprisingly, we noted a trend towards a decrease in HMGB1 concentration after EP, injection of chemotherapeutics alone and ECT, although the differences were not always statistically significant.

### 3.3 Immune involvement in ECT response is tumor type and chemotherapeutic drug dependent

**Figure 5:**
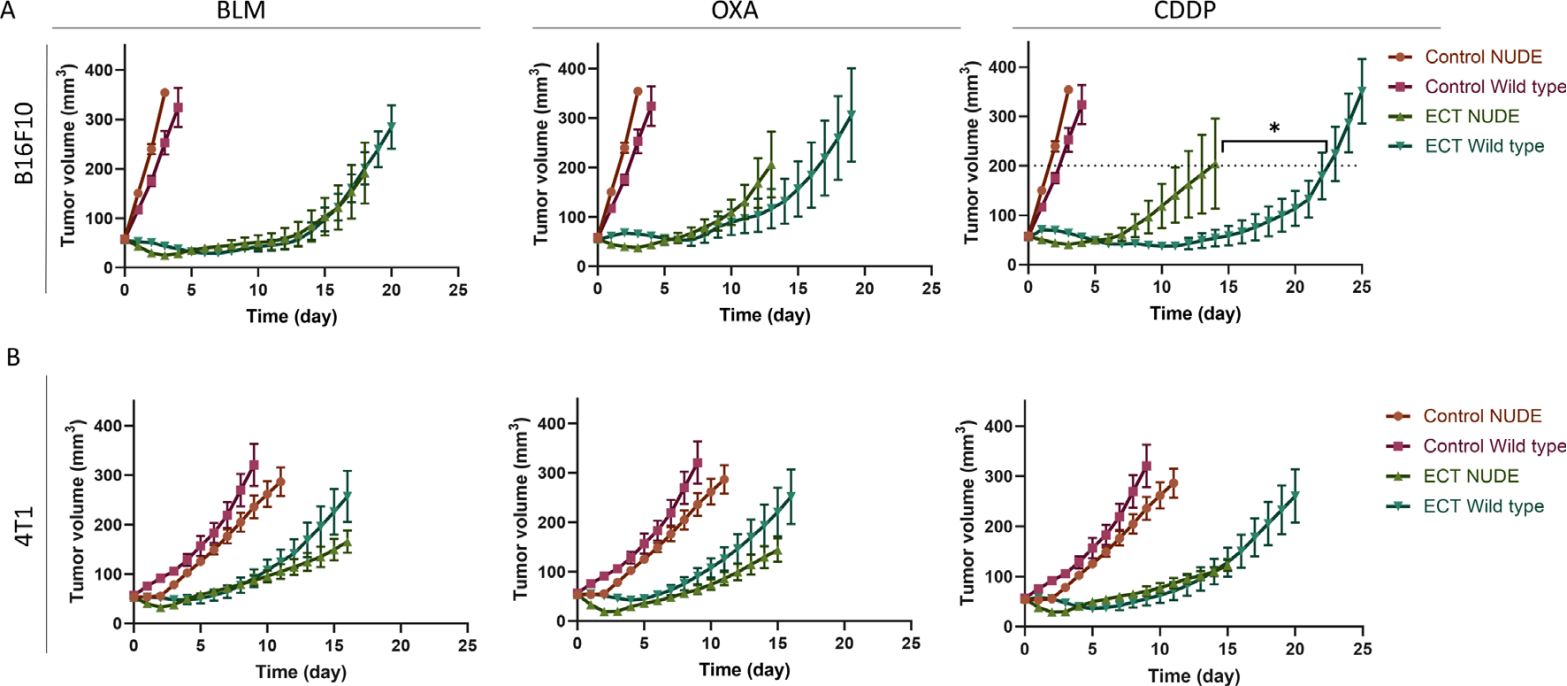
The response to ECT using CDDP is immunomodulated in mice bearing B16F10 tumors. Comparison of tumor growth after ECT in wild type and immunocompromised NUDE mice bearing **(A)** B16F10 and **(B)** 4T1 tumors. Tumor growth curves are presented (n = 6-8; AM ± SE; T test at tumor volume of 200 mm^3^; *p ≤ 0.05). Control groups for each tumor model are repeated in all the three graphs.

The utilization of immunodeficient mice allows for the investigation of immunomodulated antitumor responses. In these experiments, athymic NUDE mice, incapable of T cell production, were employed. We compared the antitumor responses to ECT with BLM, OXA, or CDDP between wild-type mice and immunodeficient mice (**Figures 5A, B**, **Supplementary Figures S7A, B**). Our findings indicate that T cells, that are impaired in immunodeficient mice, play a significant role in the antitumor response following ECT with CDDP in B16F10 melanoma. Namely, wild-type mice exhibited a better antitumor response to ECT with CDDP compared to NUDE mice. In contrast, we did not observe a significantly enhanced antitumor response to ECT with CDDP in wild-type mice bearing 4T1 tumors. This was also true for ECT with BLM or OXA in both tumor types.

### 3.4 Local tumor ablation with ECT stimulates dendritic cells

**Figure 6:**
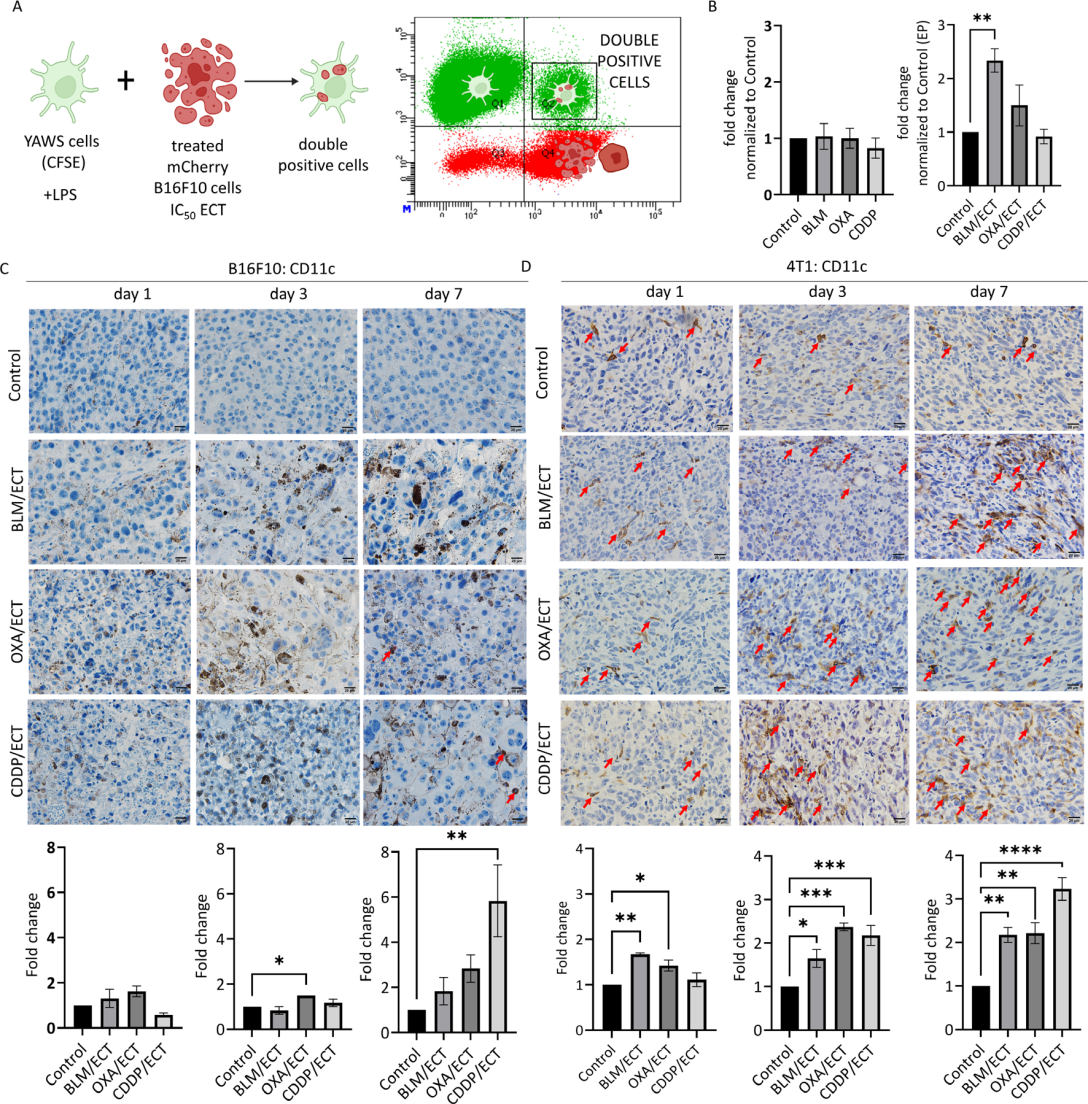
ECT stimulates dendritic cells. **(A, B)** *In vitro* phagocytic activity of dendritic cells after stimulation with ECT-treated B16F10 cells. (n=3, AM ± SE; *p ≤ 0.05, **p ≤ 0.01, ***p ≤ 0.001, ****p ≤ 0.0001). The intratumoral recruitment of dendritic cells (CD11c^+^) varies depending on the chemotherapeutic drug used in ECT, as observed in **(C)** B16F10 and **(D)** 4T1 tumors on day one, three and seven post-therapy. Arrows indicate CD11c positive dendritic cells. Scale bar: 20 µm. (n=3, AM ± SE; *p ≤ 0.05, **p ≤ 0.01, ***p ≤ 0.001, ****p ≤ 0.0001). Created with BioRender.com.

Successful *in situ* vaccination necessitates the involvement of cross-presenting dendritic cells (6). Therefore, our study focused on *in vitro* activities and *in vivo* presence of dendritic cells. Initially, to address the cross-presentation, we investigated the *in vitro* phagocytic activity of dendritic cells following stimulation with treated tumor cells. Specifically, mCherry-B16F10 tumor cells were subjected to IC_50_ doses in ECT, previously determined by Kesar et al. (18), and introduced to dendritic cells labeled with CFSE (**Figure 6A**). Cytometric analyses conducted 48 hours after -co-culturing of the two tumor types showed that only tumor cells treated with ECT using BLM successfully elevated the phagocytic activity of dendritic cells (**Figure 6B**).

Intratumoral presence of dendritic cells is crucial for efficient *in situ* vaccination due to their ability to internalize and process antigens released by tumors and thereby activate a broad and patient-specific antitumor T cell response (3). In our study, we utilized two tumor models that represent distinct immunological profiles regarding dendritic cells: the B16F10 model, characterized by minimal dendritic cell infiltrate (not exceeding 1 dendritic cell per visual field at 40× magnification), and the 4T1 model, featuring a favorable dendritic cell infiltrate (~60 dendritic cells per visual field at 40× magnification) (**Figures 6C, D**). At the initial time point, one day following the therapy, ECT failed to recruit dendritic cells in B16F10 tumor model. Furthermore, ECT attracted dendritic cells intratumorally only after ECT with OXA (day three) and ECT with CDDP (day seven). Despite a notable fold change on day seven (2 to 6-fold normalized to control), the dendritic cell infiltrate remained low (**Figure 6C**). However, in the 4T1 tumor model, ECT exhibited efficacy in attracting dendritic cells into tumors, when utilizing ECT with BLM and OXA. The fold change on days three to seven post-ECT was around 2 to 3-fold with all three chemotherapeutic drugs used (**Figure 6D**).

### 3.5 Local adaptive immune response is activated after intratumoral ECT

Although the primary aim of intratumoral treatment is local tumor ablation, the localized immune response it provokes can also be exploited systemically (3). In this context, we examined the capacity of ECT with three chemotherapeutic drugs to attract immune cells intratumorally, predisposed by the dendritic cell infiltration presented above. Particularly, we evaluated the dynamics of tumor infiltration by immune cells expressing CD4, CD8, and granzyme B (GrB).

**Figure 7:**
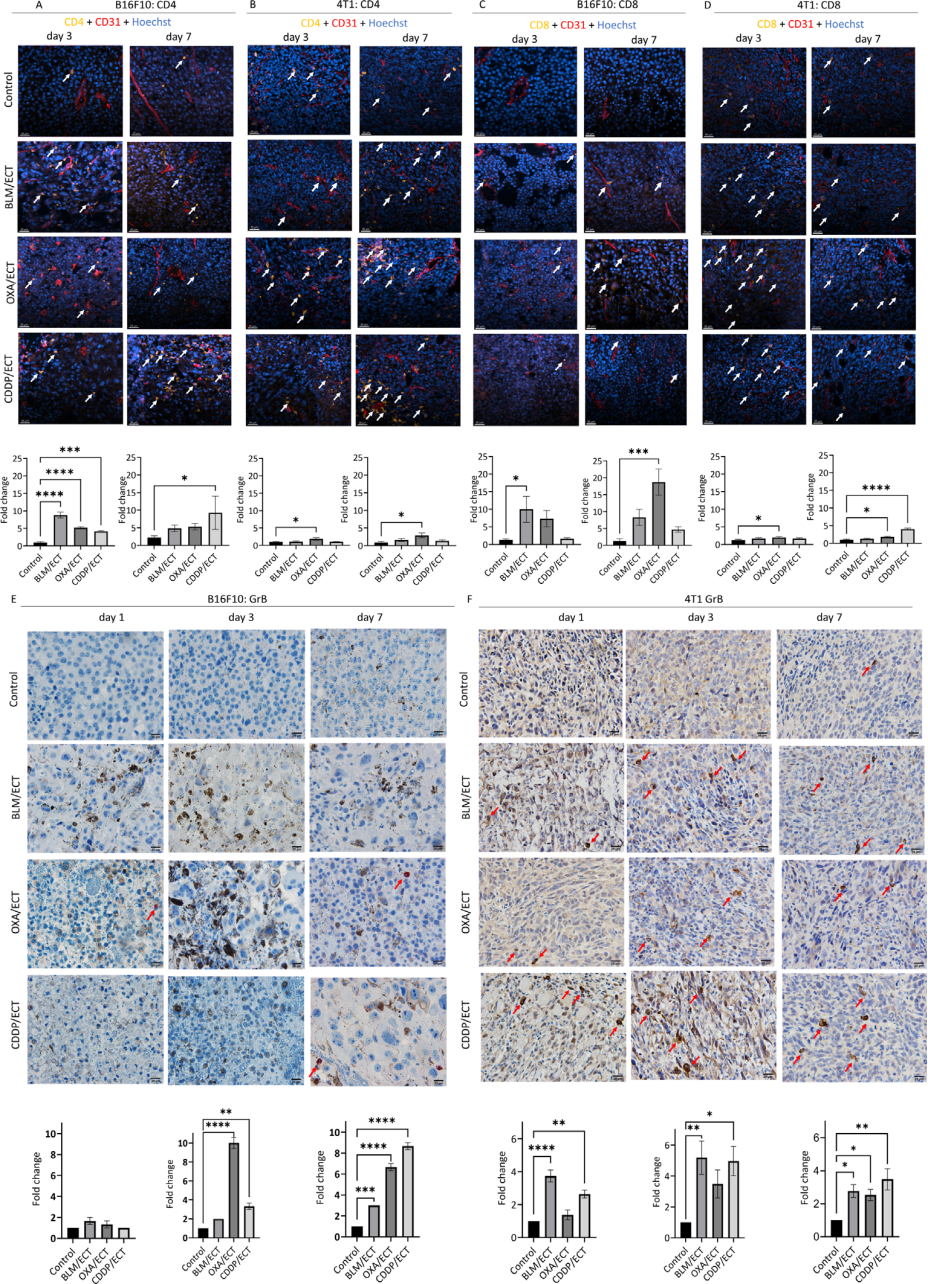
Local adaptive immune response is activated after ECT. IFC and IHC stainings of both, B16F10 and 4T1 tumors were performed one, three and seven days after the ECT with BLM, OXA or CDDP. Specifically, **(A, B)** CD4 positive immune cells, **(C, D)** CD8 positive immune cells as well as **(E, F)** granzyme B (GrB) positive effector cells were detected intratumorally in viable tumor parts. Arrows indicate IHC or IFC positive cells. Scale bar: 30 µm [IFC; **(A–D)**] and 20 µm [IHC; **(E, F)**]. (n=3, AM ± SE; *p ≤ 0.05, **p ≤ 0.01, ***p ≤ 0.001, ****p ≤ 0.0001).

Similar to the observations with dendritic cell infiltrate, B16F10 tumors displayed a lower degree of infiltration for investigated immune cell populations compared to 4T1 tumors (**Figures 7A–F**). In particular, CD4, CD8 and GrB positive immune cell populations were nearly absent in B16F10 tumors, whereas in 4T1 tumors, each cell population was represented by at least 20, 7 or 5 cells per visual field (40 × magnification), respectively. The observed difference indicates a more notable fold-change following ECT in the case of B16F10 tumors compared to 4T1 tumors. Additionally, it is worth mentioning that the assessment of immune cell populations was confined to viable regions within the B16F10 and 4T1 tumors. Therefore, extensive post-ECT necrosis significantly limited the areas relevant to detect the immune infiltrate in the tumor. This was especially notable in the case of ECT using CDDP, where the necrotic area was the most extensive (**Figure 3**).

Regarding CD4 populations in B16F10 tumors (**Figures 7A, B**), ECT with all three chemotherapeutic drugs effectively recruited CD4 positive immune cells intratumorally on day 3. In contrast, in 4T1 tumors, only ECT with OXA demonstrated efficacy in recruiting CD4 positive cells; however, on both, day three and day seven. In the case of CD8 populations (**Figures 7C, D**), in B16F10 tumors, ECT with BLM (day three) and OXA (day seven) proved effective in recruiting immune cells. Additionally, successful recruitment was also observed in 4T1 tumors, specifically after ECT with OXA (day three and day seven) as well as with CDDP (day seven). Finally, effector immune cells expressing GrB, such as natural killer cells and cytotoxic T cells (36), were assessed on days one, three, and seven following ECT (**Figures 7E, F**). Despite varied infiltration on days one and three post-ECT, by day seven ECT with all three chemotherapeutic drugs successfully attracted effector cells intratumorally in both tumor models.

### 3.6 Implication of systemic antitumor effectiveness after intratumoral ECT

**Figure 8:**
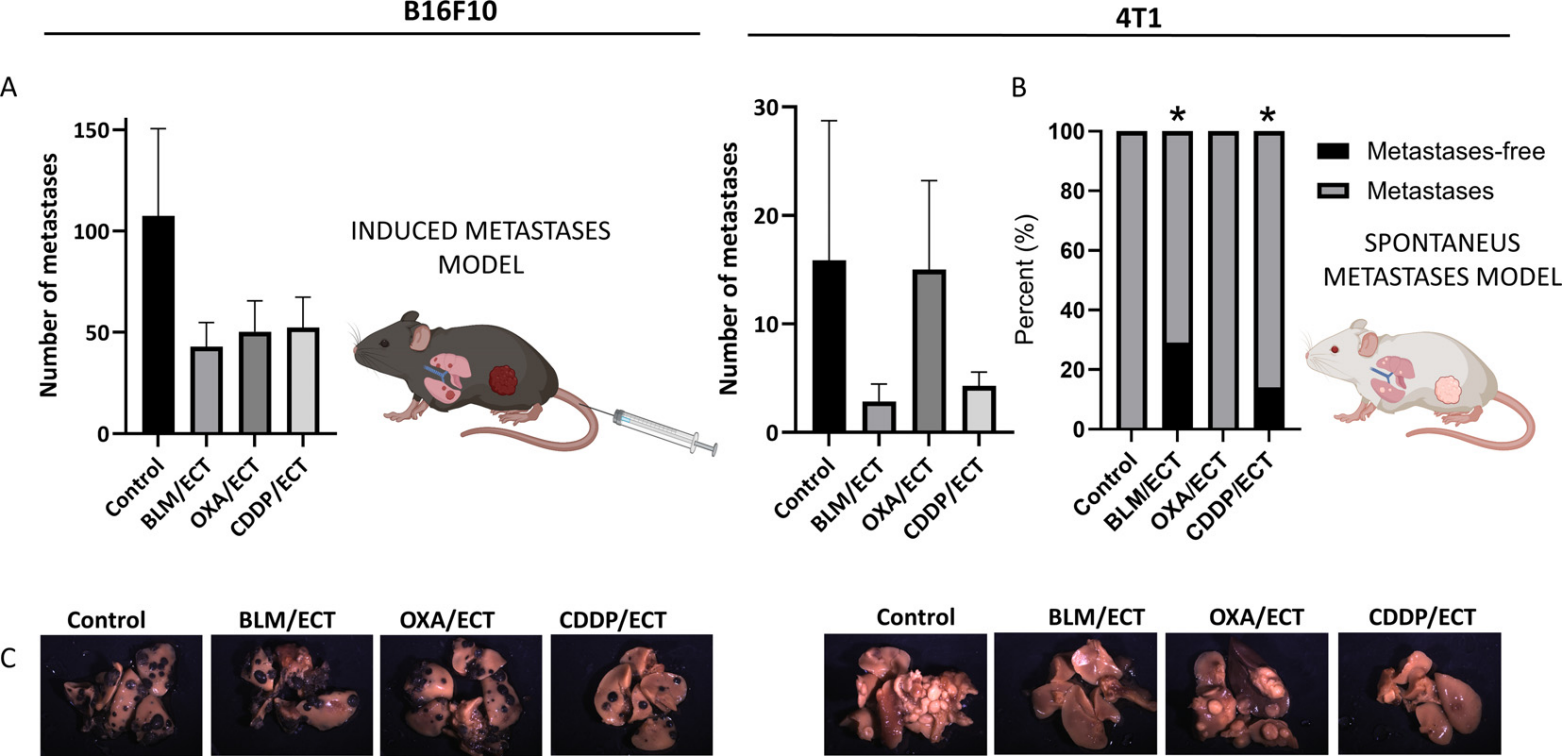
Systemic antitumor effectiveness after intratumoral ECT. **(A)** Two mouse models of systemic disease were used; B16F10 mouse metastatic model established through induced lung metastases and 4T1 spontaneous metastases model (n=6-8; AM ± SE). **(B)** Percentage of mice without detected metastasis at human endpoint in 4T1 spontaneous metastases model (two-sided Fisher’s exact test, *p ≤ 0.0001). **(C)** Representative images of mouse lungs of untreated mice and mice post-ECT. Created with BioRender.com.

In exploring the systemic antitumor effects or protective capabilities of local therapies, mouse metastases models are indispensable in preclinical cancer research. Here, we investigated the systemic antitumor effect of ECT with BLM, OXA or CDDP utilizing mouse B16F10 metastatic model established through induced lung metastases and 4T1 spontaneous lung metastases model (**Figure 8**). We found a trend towards reduced numbers of lung metastases following ECT with all the three chemotherapeutic drugs in the B16F10 metastasis model (**Figure 8A**). In the 4T1 model, 28.6% (2/7) and 14.3% (1/7) of mice treated with ECT/BLM and ECT/CDDP, respectively, displayed no metastases, while all mice in the control group had metastases (**Figures 8A–C**).

## 4 Discussion

In this study, we examined the effectiveness of intratumoral ECT with BLM, OXA and CDDP. Our objective was to conduct a comprehensive comparison of both direct cytotoxic effects and, notably, immunologically significant events resulting from intratumoral ECT with three distinct chemotherapeutic drugs. To achieve this, we employed two immunologically cold tumor models—B16F10 melanoma and 4T1 mammary carcinoma. Our findings indicate that intratumoral ECT, beyond its direct cytotoxic impact, stimulates adaptive immune system locally with all three chemotherapeutic drugs. Furthermore, our study demonstrates systemic immune activation, as ECT with BLM and CDDP in 4T1 tumor model resulted in an abscopal effect.

Systemic anticancer treatments offer a comprehensive approach to address malignancies throughout the body. The systemic delivery of therapeutic agents, such as chemotherapeutic drugs, can result in widespread impact on both malignant and healthy tissues, leading to a spectrum of side effects that often pose a considerable burden on patients’ body (37). Local ablative therapies, on the other hand, target only the primary tumor, but can also play a crucial role in modulating the immune response. By stimulating the immune system locally, these therapies hold the potential to induce an abscopal effect—a phenomenon where the immune response is activated not only at the treated site but also at distant, untreated tumor sites. This dual impact represents a potential for a more comprehensive and systemic antitumor response.

The specific modality within local ablative therapies is intratumoral ECT. The application of electric pulses to the tumor site in conjunction with reduced chemotherapeutic doses aims to maximize therapeutic efficacy while minimizing systemic exposure to cytotoxic agents (38). Moreover, the antitumor activity of ECT is not limited to chemical killing effects, but also extends to immunomodulatory actions (8, 17, 18, 23, 39).

To quantify the contribution of both cytotoxic impact and immune activity in the antitumor response to ECT, establishing equieffective ECT with BLM, OXA, or CDDP was a fundamental prerequisite for comprehensively exploring the underlying mechanisms in our study. Our specific objective was to assess and compare the background mechanisms that result in equieffective ECT across two tumor models. Consistent with previous observations (8), in the 4T1 tumor model significantly lower chemotherapeutic doses in ECT were required to achieve equieffectiveness. Notably, the *in vitro* responsiveness of 4T1 tumor cells to ECT was shown to be comparable to the response of B16F10 tumor cells (8, 18), suggesting the involvement of other mechanisms *in vivo*.

The type of cell death following therapies plays a crucial role in initiating immunologically significant events, and the antitumor immune response, triggered by the death of tumor cells, contributes significantly to disease control (40). Even electric pulses alone (31, 41, 42) or chemotherapy alone (43) can trigger these events. Following ECT with BLM, OXA or CDDP, documented outcomes include apoptosis, necrosis, and immunogenic cell death (17, 18, 31, 44). Furthermore, the mechanisms of cell deaths were closely associated with the extent of internalized chemotherapeutic drugs (45). In the realm of immunogenic, tolerogenic, and silent cell deaths (19), our specific focus here was on immunologically recognizable cell deaths, namely necrosis and immunogenic cell death. It is noteworthy that our study represents the first comparison of the immunologically recognizable types of cell death in preclinical *in vivo* models with intratumoral ECT between the three chemotherapeutics BLM, OXA and CDDP.

The application of intratumoral ECT with BLM, OXA, or CDDP resulted in a noticeable increase in necrotic areas in both tumor models. Interestingly, the degree of necrosis was more pronounced in the B16F10 tumor model than in the 4T1 tumor model. This distinction became particularly evident seven days after the ECT. Hence, this confirms the involvement of additional mechanisms that contribute to equieffectiveness of intratumoral ECT such as immunological component. We confirmed the immunogenic cell death after electrochemotherapy with all the three chemotherapeutic drugs. Namely, CLR translocation and HMGB1 release, markers of immunogenic cell death, were detected after ECT in both tumor models. This result is consistent with our previous *in vitro* study on B16F10 and 4T1 tumor models (18) that demonstrated that ECT with all three tested chemotherapeutics induced ICD-associated DAMPs in a cell line and chemotherapeutic concentration specific- manner. Since quantification was extensively studied *in vitro* in the mentioned study, our aim here was solely to confirm the occurrence of immunogenic cell death *in vivo*. Our results suggest that immunologically recognizable cell deaths, necrosis and ICD, are evident as early as 24 hours post ECT.

To upgrade our investigation of kinetic changes and make comparisons based on tumor cell type, chemotherapeutic drug type, and dosage, examination of cell death mechanisms within first 24 hours after ECT would be more informative. Namely, the previous studies (44) indicate both direct necrotic cell damage and a rapid activation of apoptotic mechanisms, detectable as early as 10 minutes post-ECT, peaking at 3 hours post-ECT, and persisting in the subsequent days. The apoptotic phase precedes extensive tumor necrosis at 48–72 hours post-ECT. Therefore, investigating the kinetic differences in cell death mechanisms specific to the chemotherapeutic drug in ECT requires examination within hours post-ECT.

Given this context, there is a demand for personalized therapeutic approaches and the identification of biomarkers to tailor therapy (de)escalation during the course of ECT (38). Blood-based biomarkers appear promising due to their ease of collection and the ability to be repeatedly measured during treatment. Serum HMGB1 concentration appears to reflect complex and diverse immunogenic responses (46). Apart from serving as an indicator of immunogenic cell death, extracellular HMGB1 has consistently been utilized as a biomarker for general plasma membrane permeabilization (47). Additionally, HMGB1 could be employed as a diagnostic marker for early cancer detection. Specifically, HMGB1 levels are elevated in cancer patients, both locally in tumor cells and systemically in serum (48, 49). Additionally, infection and treatment-associated toxicity should be considered when interpreting the dynamics of HMGB1 (50). As these events are interconnected, it is challenging to pinpoint which event or combination of events is responsible for the altered HMGB1 concentration in tumor-bearing mice and treated mice.

In this paper, we investigated serum HMGB1 concentrations in naïve, untreated, and ECT-treated mice. ELISA tests on serum confirmed that tumor-bearing mice have higher blood HMGB1 concentrations compared to naïve mice. However, we observed a trend toward decreased serum HMGB1 concentration post-ECT. Yet, the decrease was not consistent and depended on the tumor type and chemotherapeutic drug. Our results suggest that serum HMGB1 concentration primarily reflects tumor burden, which, as described above, is a potential confounder and therefore may not be a reliable biomarker of response to ECT. However, HMGB1 increase was evident locally at the tumor site after ECT, while it was undetectable in untreated tumors. Therefore, we showed that this DAMP is present after ECT at the treatment site, confirming the ECT’s potential for *in situ* vaccination.

The utilization of immunodeficient mice, such as athymic NUDE mice, incapable of T cell production, enabled us the examination of immune-modulated antitumor responses. The initial studies that first demonstrated the enhanced response to ECT with CDDP and BLM in wild-type mice compared to immunodeficient mice were conducted already in the 1990s (39, 51). Nevertheless, the strength of our study lies in the fact that we conducted a comparative analysis of intratumoral ECT using BLM, OXA, and CDDP within a single study.

We observed a significant role of T cells in the antitumor response in B16F10 melanoma following ECT with CDDP. Unexpectedly, the absence of a differential antitumor response with the other two chemotherapeutic drugs administered was notable in both the B16F10 tumor model and the 4T1 tumor model. However, this could be attributed to suboptimal doses of chemotherapeutic drugs in ECT used in the current study, compared to our previous study (39), where the differential response was apparently evident in the fraction of complete responses. Specifically, immune system responses particularly play a crucial role in eliminating the minority of remaining tumor cells post-therapy. Therefore, the predominance of cytotoxic effects over the immunological effects may contribute to these outcomes. Recognizing that the ablation potential could obscure the immunological effects, we hypothesized that these effects remain significant. To investigate this further, histological analyses of the tumor infiltrate were subsequently conducted. Furthermore, it is important to note that T cells are not the sole immune cell populations involved in the immune-antitumor response post-ECT. Therefore, utilizing other immunodeficient mouse strains or implementing strategies to selectively block specific immune cell populations (52) would provide a more precise understanding of the immune involvement.

The aim of intratumoral immunological treatments is to provoke a localized immune response, exploiting its potential to identify and combat both nearby and distant tumors (3, 7, 53). Initially, post-therapy immunostimulatory tumor microenvironment, including immunologically important changes on tumor cells as well as immunologically recognizable ways of cell death accompanied by DAMPs release (8, 18) attract dendritic cells intratumorally. Thereafter, *in situ* vaccination requires priming of CD8+ T cell responses against solid tumors and is predisposed by the involvement of cross-presenting specialized dendritic loaded with tumor antigens (5, 53, 54). Therefore, to examine that, our study firstly focused on *in vitro* and *in vivo* activities of dendritic cells after ECT.

Through *in vitro* phagocytic experiments, we demonstrated that B16F10 tumor cells treated by ECT using BLM effectively enhanced the phagocytic activity of dendritic cells 48 hours after co-culturing. However, this stimulation was not observed with chemotherapy alone or in any other ECT group. One possible mechanism for dendritic cell activation is through an HMGB1-dependent manner (6). Since it has been confirmed that the expression of HMGB1 is elevated after ECT, this could be one of the potential mechanisms. However, the kinetic differences in HMGB1 release, as assessed in our previous *in vitro* study (18), cannot fully account for the variations in phagocytic activity stimulation among the different ECT groups utilizing various chemotherapeutic drugs.

A limitation of the conducted experiment was that only one time point after the therapy was assessed. Furthermore, due to the different growth media of tumor cells and dendritic cells, only a portion of the tumor cell medium was introduced to dendritic cells with soluble factors released from dying tumor cells. The sole rationale that could be inferred for the chosen time point after ECT, specifically 48 hours post-therapy, is that immunostimulatory DAMPs following ECT using BLM may occur more rapidly compared to ECT with OXA or CDDP. However, further studies are necessary to investigate and address this hypothesis.

Intratumoral presence of dendritic cells is crucial for efficient *in situ* vaccination due to their ability to internalize and process antigens released by tumors and thereby activate a broad and patient-specific antitumor T cell response. (3, 55). The initial state of high dendritic cell infiltration was shown to correlate with the “inflamed phenotype” of the tumor immune status rather than tumor mutational burden, suggesting that the limitation in priming antitumor T cells is more associated with neoantigen presentation than neoantigen expression (56–58). This is important considering the tumor models used in our study: namely 4T1 tumors have low mutational burden but high MHC-1 expression compared to B16F10 with a higher mutational burden but low MHC-1 expression (8, 32–34). In our study, we observed variations in dendritic cell infiltration, with 4T1 tumors exhibiting higher infiltration compared to B16F10 tumors. In fact, using ECT as a strategy for *in situ* vaccination, one of our aims was to address the challenge of low intratumoral dendritic cell numbers.

There is only one clinical study that examines dendritic cell subsets at the lesion site both before and after ECT (59). The study demonstrated effective infiltration of dendritic cells in and around the melanoma lesions undergoing ECT with BLM. In our study, ECT demonstrated greater success in attracting dendritic cells in the 4T1 tumor model compared to B16F10 tumor model, regardless of the chemotherapeutic drug used. We thus speculate that initial dendritic cell status correlates with the higher immune-modulated antitumor response. Given that the doses in ECT were equieffective for B16F10 and 4T1 tumors in our case, we propose that the immunological component in the antitumor response is more pronounced in 4T1 tumors compared to B16F10 tumors. Another piece of evidence supporting this hypothesis is the lower necrotic area observed in 4T1 tumors following ECT, as described above. In our study, we did not consider the activation state of dendritic cells. Namely, dendritic cells are immunogenic when activated but tolerogenic when immature (60). Therefore, for the future studies, we propose to take into the account both, (strong) dendritic cells activation stimuli and dendritic cell activation markers after ECT.

To prove *in situ* vaccination, we further examined the capacity of ECT with the three chemotherapeutic drugs to attract immune cells intratumorally, predisposed by the previously discussed dendritic cell infiltration. Specifically, we assessed the dynamics of tumor infiltration by immune cells, focusing on adaptive immunity markers CD4, CD8, and GrB. In line with previous reports (8, 32), B16F10 tumors exhibited lower infiltration levels of the examined immune cell populations compared to 4T1 tumors. ECT demonstrated success in recruiting CD4, CD8, and GrB-positive immune cells intratumorally. The kinetics and extent of the tumor immune infiltrate positive for CD4 and CD8 following ECT were contingent upon both: the chemotherapeutic drug used and the type of tumor. Our findings align with preclinical and clinical studies on ECT and immune infiltrate (44, 61–63). Notably, CD4 and CD8 T lymphocytes were detected at all stages of the tissue reaction following ECT with BLM. In the study by Bigi et al. (44), the post-ECT infiltrate was primarily composed of CD8+ cells, with CD4+ lymphocytes being scarce. Similarly, in the investigation by Di Gennaro et al. (62), CD8+ T cells increased in the perilesional dermis post-ECT, but were rare at the tumor border and within the lesion, with no significant variation in CD4+ T cell numbers. It is important to note that CD4-positive lymphocytes play a multifaceted role in the anti-tumor immune response, since CD4 marker is also expressed in regulatory T cells with immunosuppressive characteristics (64), a phenomenon also observed after ECT (62).

In our study, mice exhibited non-uniform changes in the immunological microenvironment post-ECT, with some showing robust infiltration and others only a moderate, consistent with other studies (6, 62). This variability may partially explain the ineffectiveness of the combination of ECT and immunotherapy in some patients, as sufficient T cell infiltration in tumor tissues is a prerequisite for responding to immunotherapies (65). We assume that substantial post-ECT necrosis at days one and three post-ECT significantly restricted areas representing the tumor immune infiltrate. Again, this observation aligns with findings from clinical studies following ECT with BLM (44, 62). In our study, and the aforementioned investigations, the infiltrate was notably concentrated at the tumor margin, around the central necrosis, or in close proximity to dead tumor cells – key elements in the late inflammatory response.

Among the three investigated immune cell populations, our focus centered on GrB-positive effector cells, encompassing natural killer cells and cytotoxic T cells, serving as a biomarker indicative of immune priming (36). Significantly, by day seven post-ECT, ECT with all three chemotherapeutic drugs successfully attracted effector cells intratumorally in both tumor models. This underscores the induction of specific adaptive immune responses, collectively indicating successful *in situ* vaccination.

Collectively, our data demonstrate that comparing immunologically significant events following ECT with the three chemotherapeutic drugs in two tumor models is complex and challenging. This complexity is particularly apparent when making comparisons across time points. Notably, the similar antitumor responses observed after ECT in both immunocompromised and wild type mice do not inherently exclude the involvement of an antitumor immune response.

In this study, we exclusively focused on adaptive immune responses. However, the antitumor immunity encompasses both innate and adaptive immune responses, both contributing to tumor control (66, 67). Considering the available data, it is crucial not to overlook the involvement of innate responses in the antitumor response, even after pulsed electric fields. For instance, in a clinical study on melanoma, clusters of CD56+ natural killer cells were observed within tumor nodules, appearing as early as 3 hours and persisting up to 1 month after ECT with BLM (44).

Finally, to substantiate the systemic antitumor effect of intratumoral ECT with BLM, OXA, or CDDP, we employed mouse metastatic models. Notably, rare instances of an abscopal effect have been reported in previous preclinical models following ECT monotherapy. These cases involved contralateral tumors representing treated tumors and untreated distant metastases, specifically: intratumoral ECT with BLM (8, 17) and CDDP (22) in CT26 colon carcinoma, intratumoral ECT with BLM or CDDP in B16F10 melanoma (8), and ECT with BLM in 4T1 mammary carcinoma (23). In the latter tumor model, BLM was administered systemically. ECT with intratumoral CDDP also reduced metastatic tumor burden in the lung in the Lewis lung carcinoma tumor model (22).

In the current study, the systemic effect of intratumoral ECT was examined in lung metastases, including both spontaneous (4T1) and induced (B16F10) models. Mice within the same treatment group showed varying responses, resulting in a wide range of outcomes. Although not statistically significant, there was a trend towards fewer lung metastases following ECT with all three chemotherapeutics in the B16F10 metastatic model. It is worth noting that, in the current study, we used suboptimal doses of chemotherapeutics to enable future combination with immunotherapies, which may explain the lack of statistically significant differences in the B16F10 tumor model. Nonetheless, despite the suboptimal chemotherapeutic doses, our findings suggest a systemic induction of the immune response and confirm the presence of an abscopal effect in the 4T1 tumor metastatic model following ECT with BLM and CDDP. Notably, a subset of mice in these groups exhibited a complete absence of metastases.

In conclusion, this is the first preclinical study delving into the dual impact of intratumoral ECT using three distinct chemotherapeutic drugs – BLM, OXA, and CDDP – using two immunologically cold mouse tumor models. Our findings confirmed the involvement of the adaptive immune system in the antitumor response for all three variations of ECT. Differences in immune intervention after equieffective intratumoral ECT were highlighted by variable kinetics of immunologically recognizable cell deaths and immune infiltrate across the studied tumor models. Particularly, the 4T1 tumor model exhibited a more pronounced involvement of the immune component compared to the B16F10 tumor model. Nevertheless, in both cases, ECT demonstrated effectiveness in inducing *in situ* vaccination; however, an abscopal effect was observed in the 4T1 tumor model only. The deciphered variable kinetics and antitumor (immune) response to ECT across different tumor models and chemotherapeutic drugs lay the foundation for further investigations. This knowledge aims to strategize and implement combined treatments more effectively in future studies.

## Data Availability

The original contributions presented in the study are included in the article/**Supplementary Material**. Further inquiries can be directed to the corresponding author.

## References

[1] Golden EB Marciscano AE Formenti S. Radiation therapy and the *in situ* vaccination approach. C. Int J Radiat Oncol Biol Phys. (2020) 108:891–8. doi: 10.1016/J.IJROBP.2020.08.023 32800803

[2] Elizabeth Nelson B Adashek JJ Lin SH Subbiah V. The abscopal effect in patients with cancer receiving immunotherapy. Med. (2023) 4:233–44. doi: 10.1016/j.medj.2023.02.003 PMC10116408 36893753

[3] Pierce RH Campbell JS Pai SI Brody JD Kohrt HEK. *In-situ* tumor vaccination: Bringing the fight to the tumor. Hum Vaccin Immunother. (2015) 11:1901–9. doi: 10.1080/21645515.2015.1049779 PMC4635874 26055074

[4] Daud A Algazi AP Ashworth MT Fong L Lewis J Chan SE. Systemic antitumor effect and clinical response in a phase 2 trial of intratumoral electroporation of plasmid interleukin-12 in patients with advanced melanoma. J Clin Oncol. (2014) 32:9025–5. doi: 10.1200/jco.2014.32.15_suppl.9025

[5] Hammerich L Bhardwaj N Kohrt HE Brody JD. *In situ* vaccination for the treatment of cancer. Immunotherapy. (2016) 8:315–30. doi: 10.2217/imt.15.120 26860335

[6] Hammerich L Marron TU Upadhyay R Svensson-Arvelund J Dhainaut M Hussein S. Systemic clinical tumor regressions and potentiation of PD1 blockade with *in situ* vaccination. Nat Med. (2019) 25:814–24. doi: 10.1038/s41591-019-0410-x 30962585

[7] Sersa G Teissie J Cemazar M Signori E Kamensek U Marshall G. Electrochemotherapy of tumors as *in situ* vaccination boosted by immunogene electrotransfer. Cancer Immunology Immunotherapy. (2015) 64:1315–27. doi: 10.1007/s00262-015-1724-2 PMC4554735 26067277

[8] Ursic K Kos S Kamensek U Cemazar M Miceska S Markelc B. Potentiation of electrochemotherapy effectiveness by immunostimulation with IL-12 gene electrotransfer in mice is dependent on tumor immune status. J Control Release. (2021) 332:623–35. doi: 10.1016/J.JCONREL.2021.03.009 33705828

[9] Campana LG Daud A Lancellotti F Arroyo JP Davalos RV Di Prata C. Pulsed electric fields in oncology: A snapshot of current clinical practices and research directions from the 4th world congress of electroporation. Cancers (Basel). (2023) 15. doi: 10.3390/CANCERS15133340 PMC10340685 37444450

[10] Marty M Sersa G Garbay JR Gehl J Collins CG Snoj M. Electrochemotherapy – An easy, highly effective and safe treatment of cutaneous and subcutaneous metastases: Results of ESOPE (European Standard Operating Procedures of Electrochemotherapy) study. Eur J Cancer Suppl. (2006) 4:3–13. doi: 10.1016/j.ejcsup.2006.08.002

[11] Campana LG Edhemovic I Soden D Perrone AM Scarpa M Campanacci L. Electrochemotherapy e Emerging applications technical advances, new indications, combined approaches, and multi-institutional collaboration. Eur J Surg Oncol. (2019) 45:92–102. doi: 10.1016/j.ejso.2018.11.023. 30528893

[12] Mir LM Gehl J Sersa G Collins CG Garbay JR Billard V. Standard operating procedures of the electrochemotherapy: Instructions for the use of bleomycin or cisplatin administered either systemically or locally and electric pulses delivered by the CliniporatorTM by means of invasive or non-invasive electrodes. Eur J Cancer Supplement. (2006) 4:14–25. doi: 10.1016/j.ejcsup.2006.08.003

[13] Gehl J Sersa G Matthiessen LW Muir T Soden D Occhini A. Updated standard operating procedures for electrochemotherapy of cutaneous tumours and skin metastases. Acta Oncol (Madr). (2018) 57:874–82. doi: 10.1080/0284186X.2018.1454602 29577784

[14] Markelc B Sersa G Cemazar M. Differential mechanisms associated with vascular disrupting action of electrochemotherapy: intravital microscopy on the level of single normal and tumor blood vessels. PloS One. (2013) 8:59557. doi: 10.1371/journal.pone.0059557 PMC3608732 23555705

[15] Serša G Cemažar M Markelc B. Blood flow modifying and vascular-disrupting effects of electroporation and electrochemotherapy. Handb Electroporation. (2017) 1:691–705. doi: 10.1007/978-3-319-32886-7_165/COVER

[16] Zmuc J Gasljevic G Sersa G Edhemovic I Boc N Seliskar A. Large liver blood vessels and bile ducts are not damaged by electrochemotherapy with bleomycin in pigs. Sci Rep. (2019) 9:3649. doi: 10.1038/s41598-019-40395-y 30842517 PMC6403381

[17] Calvet CY Famin D André FM Mir LM. Electrochemotherapy with bleomycin induces hallmarks of immunogenic cell death in murine colon cancer cells. Oncoimmunology. (2014) 3:e28131. doi: 10.4161/onci.28131 25083316 PMC4111940

[18] Kesar U Markelc B Jesenko T Valentinuzzi KU Cemazar M Strojan P. Effects of electrochemotherapy on immunologically important modifications in tumor cells. Vaccines. (2023) 11:925. doi: 10.3390/VACCINES11050925 37243029 PMC10223734

[19] Green DR Ferguson T Zitvogel L Kroemer G. Immunogenic and tolerogenic cell death. Nat Rev Immunol. (2009) 9:353–63. doi: 10.1038/NRI2545 PMC2818721 19365408

[20] Kroemer G Galluzzi L Kepp O Zitvogel L. Immunogenic Cell Death in Cancer Therapy ICD: immunogenic cell death. Annu Rev Immunol. (2013) 31:51–72. doi: 10.1146/annurev-immunol-032712-100008 23157435

[21] Kamensek U Kos S Sersa G. Adjuvant immunotherapy as a tool to boost effectiveness of electrochemotherapy. Handb Electroporation. (2016), 1–16. doi: 10.1007/978-3-319-26779-1_105-2

[22] Tremble LF O’Brien MA Soden DM Forde PF. Electrochemotherapy with cisplatin increases survival and induces immunogenic responses in murine models of lung cancer and colorectal cancer. Cancer Lett. (2019) 442:475–82. doi: 10.1016/j.canlet.2018.11.015 30472183

[23] Ruzgys P Navickaitė D Palepšienė R Uždavinytė D Barauskaitė N Novickij V. Induction of bystander and abscopal effects after electroporation-based treatments. Cancers (Basel). (2022) 14. doi: 10.3390/CANCERS14153770 PMC9367330 35954434

[24] Goggins CA Khachemoune A. The use of electrochemotherapy in combination with immunotherapy in the treatment of metastatic melanoma: a focused review. Int J Dermatol. (2019) 58:865–70. doi: 10.1111/IJD.14314 30479009

[25] Justesen TF Orhan A Raskov H Nolsoe C Gögenur I. Electroporation and immunotherapy—Unleashing the abscopal effect. Cancers (Basel). (2022) 14. doi: 10.3390/CANCERS14122876 PMC9221311 35740542

[26] Hadzialjevic B Omerzel M Trotovsek B Cemazar M Jesenko T Sersa G. Electrochemotherapy combined with immunotherapy – a promising potential in the treatment of cancer. Front Immunol. (2024) 14:1336866. doi: 10.3389/FIMMU.2023.1336866 38292489 PMC10825954

[27] Lampreht Tratar U Milevoj N Cemazar M Znidar K Ursic Valentinuzzi K Brozic A. Treatment of spontaneous canine mast cell tumors by electrochemotherapy combined with IL-12 gene electrotransfer: Comparison of intratumoral and peritumoral application of IL-12. Int Immunopharmacol. (2023) 120:110274. doi: 10.1016/j.intimp.2023.110274 37216797

[28] Tellado M De Robertis M Montagna D Giovannini D Salgado S Michinski S. Electrochemotherapy plus IL-2+IL-12 gene electrotransfer in spontaneous inoperable stage III-IV canine oral Malignant melanoma. Vaccines (Basel). (2023) 11. doi: 10.3390/VACCINES11061033 PMC10301420 37376422

[29] Heppt MV Eigentler TK Kähler KC Herbst RA Göppner D Gambichler T. Immune checkpoint blockade with concurrent electrochemotherapy in advanced melanoma: a retrospective multicenter analysis. Cancer Immunology Immunotherapy. (2016) 65:951–9. doi: 10.1007/s00262-016-1856-z PMC11029138 27294607

[30] Campana LG Peric B Mascherini M Spina R Kunte C Kis E. Combination of pembrolizumab with electrochemotherapy in cutaneous metastases from melanoma: A comparative retrospective study from the inspECT and Slovenian cancer registry. *Cancers (Basel)*. (2021) 13:4289. doi: 10.3390/cancers13174289 PMC8428335 34503099

[31] Ursic K Kos S Kamensek U Cemazar M Scancar J Bucek S. Comparable effectiveness and immunomodulatory actions of oxaliplatin and cisplatin in electrochemotherapy of murine melanoma. Bioelectrochemistry. (2018) 119:161–71. doi: 10.1016/j.bioelechem.2017.09.009 29024870

[32] Lechner MG Karimi SS Barry-Holson K Angell TE Murphy KA Church CH. Immunogenicity of murine solid tumor models as a defining feature of *in vivo* behavior and response to immunotherapy. J Immunother. (2013) 36:477–89. doi: 10.1097/01.cji.0000436722.46675.4a PMC3910494 24145359

[33] Castle JC Loewer M Boegel S Tadmor AD Boisguerin V de Graaf J. Mutated tumor alleles are expressed according to their DNA frequency. Sci Rep. (2014) 4:4743 4. doi: 10.1038/srep04743 24752137 PMC3994436

[34] Id JWY Bhattacharya S Yanamandra N Kilian D Shi H Yadavilli S. Tumor-immune profiling of murine syngeneic tumor models as a framework to guide mechanistic studies and predict therapy response in distinct tumor microenvironments. PLoS One. (2018) 13:0206223. doi: 10.1371/journal.pone.0206223 PMC6214511 30388137

[35] Sersa G Cemazar M Miklavcic D. Antitumor effectiveness of electrochemotherapy with cis-diamminedichloroplatinum(II) in mice. Cancer Res. (1995) 55:3450–5. 7614485

[36] Larimer BM Wehrenberg-Klee E Dubois F Mehta A Kalomeris T Flaherty K. Granzyme B PET imaging as a predictive biomarker of immunotherapy response. Cancer Res. (2017) 77:2318–27. doi: 10.1158/0008-5472.CAN-16-3346 PMC5474226 28461564

[37] Schirrmacher V. From chemotherapy to biological therapy: A review of novel concepts to reduce the side effects of systemic cancer treatment (Review). Int J Oncol. (2019) 54:407–19. doi: 10.3892/IJO.2018.4661 PMC6317661 30570109

[38] Sersa G Ursic K Cemazar M Heller R Bosnjak M Campana LG. Biological factors of the tumour response to electrochemotherapy: Review of the evidence and a research roadmap. Eur J Surg Oncol. (2021) 47:1836–46. doi: 10.1016/J.EJSO.2021.03.229 33726951

[39] Sersa G Miklavcic D Cemazar M Belehradek J Jarm T Mir LM. Electrochemotherapy with CDDP on LPB sarcoma: comparison of the anti-tumor effectiveness in immunocompotent and immunodeficient mice. Bioelectrochemistry Bioenergetics. (1997) 43:279–83. doi: 10.1016/S0302-4598(96)05194-X

[40] Strasser A Vaux DL. Cell death in the origin and treatment of cancer. Mol Cell. (2020) 78:1045–54. doi: 10.1016/J.MOLCEL.2020.05.014 32516599

[41] Polajzer T Jarm T Miklavcic D. Analysis of damage-associated molecular pattern molecules due to electroporation of cells *in vitro* . Radiol Oncol. (2020) 54:317–28. doi: 10.2478/RAON-2020-0047 PMC7409611 32726295

[42] Polajžer T Miklavčič D. Immunogenic cell death in electroporation-based therapies depends on pulse waveform characteristics. Vaccines (Basel). (2023) 11. doi: 10.3390/VACCINES11061036 PMC10304444 37376425

[43] Tesniere A Schlemmer F Boige V Kepp O Martins I Ghiringhelli F. Immunogenic death of colon cancer cells treated with oxaliplatin. Oncogene. (2010) 29:482–91. doi: 10.1038/onc.2009.356 19881547

[44] Bigi L Galdo G Cesinaro AM Vaschieri C Marconi A Pincelli C. Electrochemotherapy induces apoptotic death in melanoma metastases: a histologic and immunohistochemical investigation. Clin Cosmet Investig Dermatol Volume. (2016) 9:451–9. doi: 10.2147/CCID.S115984 PMC5125800 27920565

[45] Tounekti O Pron G Belehradek J Mir LM. Bleomycin, an apoptosis-mimetic drug that induces two types of cell death depending on the number of molecules internalized. Cancer Res. (1993) 53:5462–9. 7693342

[46] Fucikova J Kepp O Kasikova L Petroni G Yamazaki T Liu P. Detection of immunogenic cell death and its relevance for cancer therapy. Cell Death Dis. (2020) 11. doi: 10.1038/S41419-020-03221-2 PMC7691519 33243969

[47] Kepp O Senovilla L Vitale I Vacchelli E Adjemian S Agostinis P. Consensus guidelines for the detection of immunogenic cell death. Oncoimmunology. (2014) 3:e955691. doi: 10.4161/21624011.2014.955691 25941621 PMC4292729

[48] Wild CA Brandau S Lotfi R Mattheis S Gu X Lang S. HMGB1 is overexpressed in tumor cells and promotes activity of regulatory T cells in patients with head and neck cancer. Oral Oncol. (2012) 48:409–16. doi: 10.1016/J.ORALONCOLOGY.2011.12.009 22265157

[49] Singh A Gupta N Khandakar H Kaushal S Seth A Pandey RM. Autophagy-associated HMGB-1 as a novel potential circulating non-invasive diagnostic marker for detection of Urothelial Carcinoma of Bladder. Mol Cell Biochem. (2022) 477:493–505. doi: 10.1007/s11010-021-04299-8 34796446 PMC8601373

[50] Clasen K Welz S Faltin H Zips D Eckert F. Dynamics of HMBG1 (High Mobility Group Box 1) during radiochemotherapy correlate with outcome of HNSCC patients. Strahlentherapie und Onkologie. (2022) 198:194–200. doi: 10.1007/S00066-021-01860-8 34671818 PMC8789630

[51] Mir LM Orlowski S Belehradek J Paoletti C. Electrochemotherapy potentiation of antitumour effect of bleomycin by local electric pulses. Eur J Cancer Clin Oncol. (1991) 27:68–72. doi: 10.1016/0277-5379(91)90064-K 1707289

[52] Buqué A Galluzzi L. Modeling tumor immunology and immunotherapy in mice. Trends Cancer. (2018) 4:599–601. doi: 10.1016/J.TRECAN.2018.07.003 30149876

[53] Humeau J Le Naour J Galluzzi L Kroemer G Pol JG. Trial watch: intratumoral immunotherapy. Oncoimmunology. (2021) 10. doi: 10.1080/2162402X.2021.1984677 PMC8526014 34676147

[54] Crittenden MR Thanarajasingam U Vile RG Gough MJ. Intratumoral immunotherapy: Using the tumour against itself. Immunology. (2005) 114:11–22. doi: 10.1111/J.1365-2567.2004.02001.X 15606790 PMC1782057

[55] Dal Col J Brody J Barrio MM Castiello L Aricò E D’agostino G. *In situ* vaccination by direct dendritic cell inoculation: the coming of age of an old idea? Front Immunol. (2019) 10:2303. doi: 10.3389/fimmu.2019.02303 31611878 PMC6773832

[56] Salmon H Idoyaga J Rahman A Leboeuf M Remark R Jordan S. Expansion and activation of CD103+ dendritic cell progenitors at the tumor site enhances tumor responses to therapeutic PD-L1 and BRAF inhibition. Immunity. (2016) 44:924. doi: 10.1016/J.IMMUNI.2016.03.012 27096321 PMC4980762

[57] Sánchez-Paulete AR Cueto FJ Martínez-López M Labiano S Morales-Kastresana A Rodríguez-Ruiz ME. Cancer immunotherapy with immunomodulatory anti-CD137 and anti–PD-1 monoclonal antibodies requires BATF3-dependent dendritic cells. Cancer Discovery. (2016) 6:71–9. doi: 10.1158/2159-8290.CD-15-0510/42559/AM/CANCER-IMMUNOTHERAPY-WITH-IMMUNOMODULATORY-ANTI PMC5036540 26493961

[58] Spranger S Luke JJ Bao R Zha Y Hernandez KM Li Y. Density of immunogenic antigens does not explain the presence or absence of the T-cell-inflamed tumor microenvironment in melanoma. Proc Natl Acad Sci U.S.A. (2016) 113:E7759–68. doi: 10.1073/PNAS.1609376113 PMC5137753 27837020

[59] Gerlini G Sestini S Di Gennaro P Urso C Pimpinelli N Borgognoni L. Dendritic cells recruitment in melanoma metastasis treated by electrochemotherapy. Clin Exp Metastasis. (2013) 30:37–45. doi: 10.1007/S10585-012-9505-1/METRICS 22735940

[60] Del Prete A Salvi V Soriani A Laffranchi M Sozio F Bosisio D. Dendritic cell subsets in cancer immunity and tumor antigen sensing. Cell Mol Immunol. (2023) 20:432–47. doi: 10.1038/S41423-023-00990-6 PMC10203372 36949244

[61] Roux S Bernat C Al-Sakere B Ghiringhelli F Opolon P Carpentier AF. Tumor destruction using electrochemotherapy followed by CpG oligodeoxynucleotide injection induces distant tumor responses. Cancer Immunology Immunotherapy. (2008) 57:1291–300. doi: 10.1007/S00262-008-0462-0/METRICS PMC11031045 18259749

[62] Di Gennaro P Gerlini G Urso C Sestini S Brandani P Pimpinelli N. CD4+FOXP3+ T regulatory cells decrease and CD3+CD8+ T cells recruitment in TILs from melanoma metastases after electrochemotherapy. Clin Exp Metastasis. (2016) 33:787–98. doi: 10.1007/S10585-016-9814-X/METRICS 27475809

[63] Bendix MB Houston A Forde PF Brint E. Electrochemotherapy and immune interactions; A boost to the system? Eur J Surg Oncol. (2022) 48:1895–900. doi: 10.1016/j.ejso.2022.05.023 35667946

[64] Tay RE Richardson EK Toh HC. Revisiting the role of CD4+ T cells in cancer immunotherapy—new insights into old paradigms. Cancer Gene Ther. (2020) 28:5–17. doi: 10.1038/s41417-020-0183-x 32457487 PMC7886651

[65] Tang H Wang Y Chlewicki LK Wang J Wang X Fu Y-X. Facilitating T cell infiltration in tumor microenvironment overcomes resistance to PD-L1 blockade. Cancer Cell. (2016) 29:285–96. doi: 10.1016/j.ccell.2016.02.004 PMC4794755 26977880

[66] Mulder WJM Ochando J Joosten LAB Fayad ZA Netea MG. Therapeutic targeting of trained immunity. Nat Rev Drug Discovery. (2019) 18:553. doi: 10.1038/S41573-019-0025-4 30967658 PMC7069501

[67] McMahon RA D’Souza C Neeson PJ Siva S. Innate immunity: Looking beyond T-cells in radiation and immunotherapy combinations. Neoplasia. (2023) 46:100940. doi: 10.1016/J.NEO.2023.100940 37913654 PMC10637988

