# Supplementary material for: Electrochemotherapy with bleomycin, oxaliplatin, or cisplatin in mouse tumor models, from tumor ablation to in situ vaccination
Source: Front Immunol. 2025 Feb 11;16:1470432. doi: 10.3389/fimmu.2025.1470432 (PMC11850275; doi:10.3389/fimmu.2025.1470432)
Supplement: Supplementary file 1 [file DataSheet1.pdf]

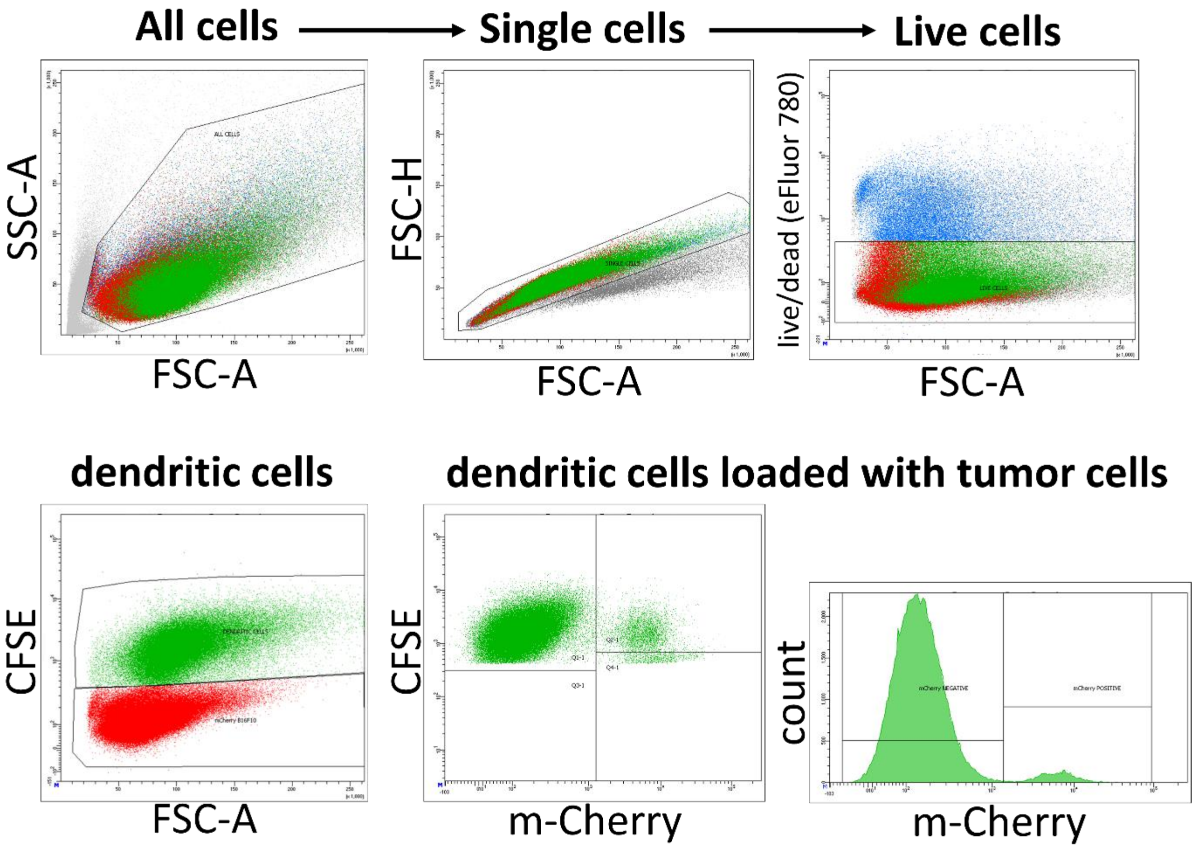

Figure S1. **Gating strategy in flow cytometry.** We conducted an assessment of the proportion of dendritic cells loaded with tumor cells. Double cells were eliminated by employing a forward scatter-area (FSC-A) and forward scatter-height (FSC-H) dot plot, and only live dendritic cells negative for eFluor 780 and positive for CFSE were considered for the evaluation of mCherry expression.

Table S1. List of antibodies. Listed are antibodies used for immunofluorescence (IFC) and immunohistochemical (IHC) staining. Antibody names, abbreviations, dilutions and manufacturers are presented.

antibody name	abbreviation	dilution	manufacturer
**Immunofluorescence staining**			
Primary: Anti CD31/PECAM-1 Goat Polyclonal Antibody AF3628	anti CD31	1:200	R&D systems
Primary: Anti-CD4 Rabbit Recombinant Monoclonal antibody EPR19514	anti CD4	1:200	Abcam
Primary: Anti CD8 alpha Rabbit Recombinant Monoclonal antibody EPR20305	anti CD8	1:200	Abcam
Primary: Anti Calreticulin Chicken Polyclonal Antibody AB_2069607	anti CLR	1:200	Invitrogen
Secondary: Donkey Anti Rabbit IgG Cy3 #711-165-152	Anti Rabbit Cy3	1: 400	Jackson Immunoresearch
Secondary: Donkey Anti Goat IgG Alexa Fluor® 647 #705-605-147	Anti Goat Alexa Fluor® 647	1: 400	Jackson Immunoresearch
Secondary: Donkey Anti-Chicken Alexa Fluor® 488 #703-545-155	Anti Chicken Alexa Fluor® 488	1: 500	Jackson Immunoresearch
**Immunofluorescence staining**			
Primary: anti HMGB1 Recombinant Rabbit Monoclonal antibody (SA39-03)	anti HMGB1	1: 500	Invitrogen
Primary: Anti Granzyme B Rabbit Polyclonal antibody ab4059	anti GrB	1: 1500	Abcam
Primary: Anti CD11c (D1V9Y) Rabbit Monoclonal Antibody #97585	anti CD11c	1:200	Cell Signaling

B16F10

A

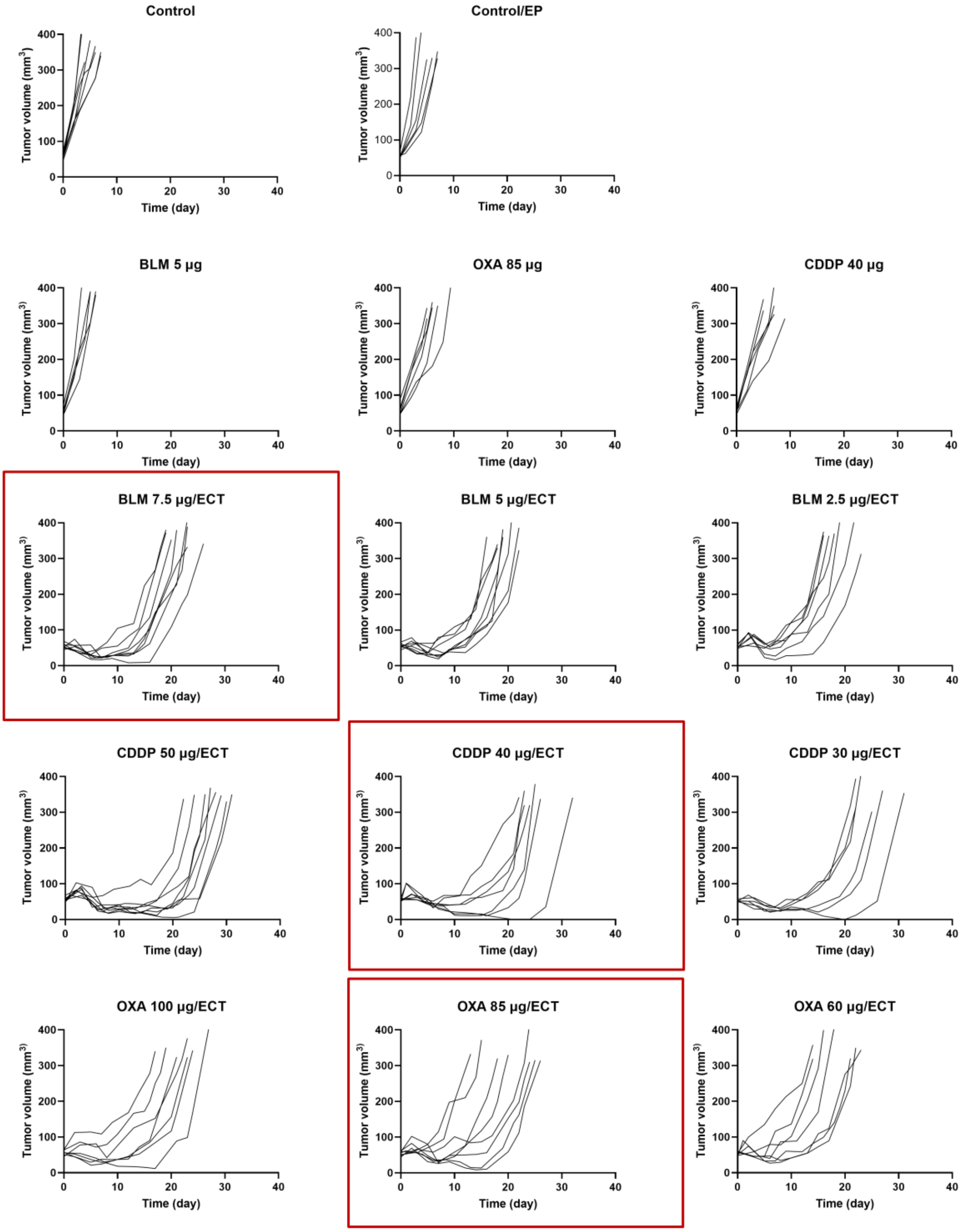
Control
Control/EP
BLM 5 µg
OXA 85 µg
CDDP 40 µg
BLM 7.5 µg/ECT
BLM 5 µg/ECT
BLM 2.5 µg/ECT
CDDP 50 µg/ECT
CDDP 40 µg/ECT
CDDP 30 µg/ECT
OXA 100 µg/ECT
OXA 85 µg/ECT
OXA 60 µg/ECT
Tumor volume (mm3)
Time (day)
0
100
200
300
400
0
10
20
30
40

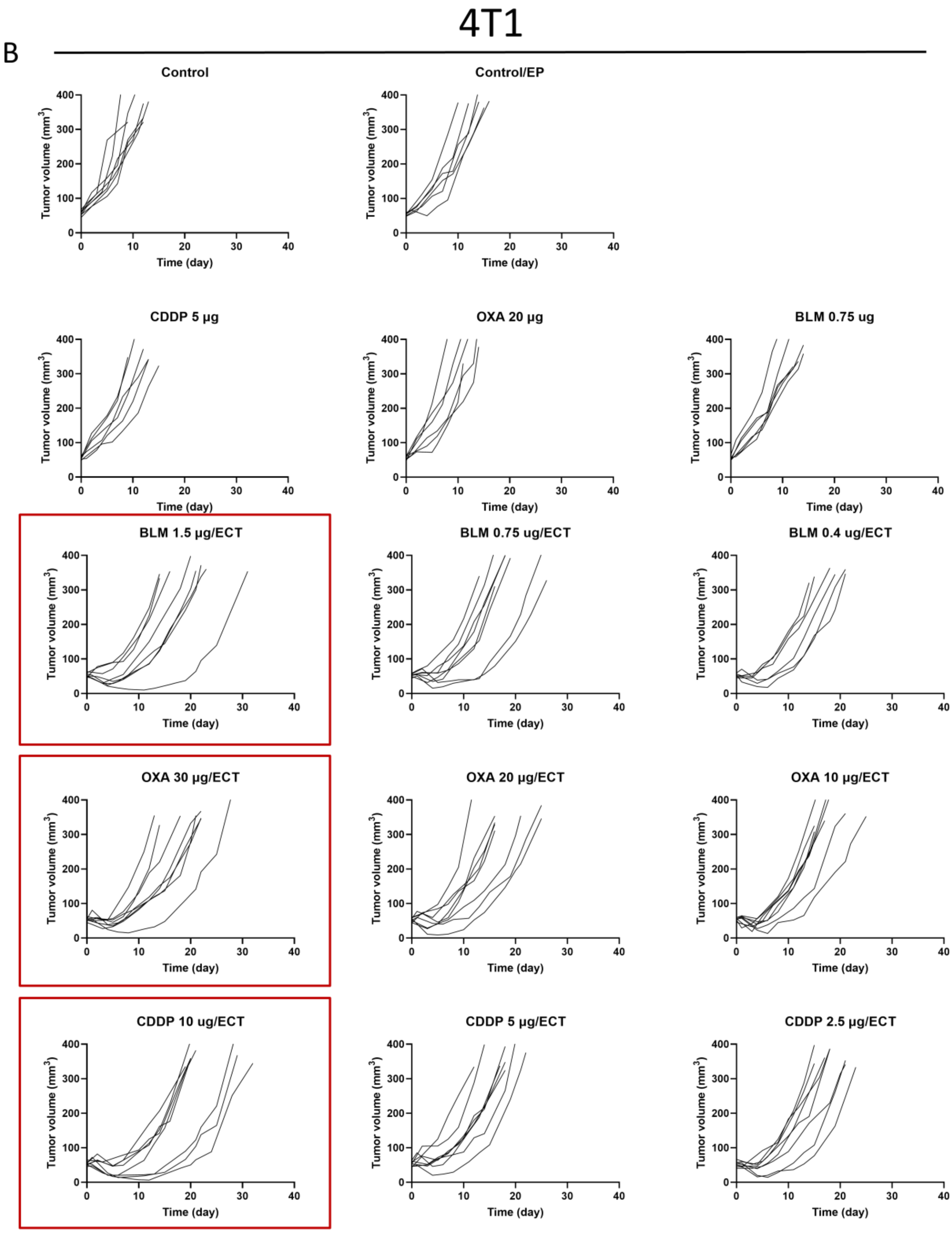

Figure S2. **Tumor growth is delayed after ECT.** Tumor growth curves of individual mice are shown for different treatment groups in **(A**) B16F10 and (**B)**, 4T1 tumors (n = 6-8). Red frames indicate equieffective ECT with BLM, OXA and CDDP or the doses in ECT, which were used for subsequent analyses. BLM: bleomycin, OXA: oxaliplatin, CDDP: cisplatin.

Table S2. Average survival is prolonged after ECT. Average survival time of treatment groups is presented. Data are compared to control group and to each other (n = 6-8; AM ± SE; *p < 0.05).

		**B16F10**		**4T1**	
Group name		V = 200 mm^3 [day]	Statistics	V = 200 mm^3 [day]	Statistics
1	Control	2.57 ± 0.25		6.67 ± 0.52	
2	Control/EP	3.63 ± 0.55		8.76 ± 0.67	
3	BLM 0.75 µg	/	/	7.06 ± 0.51	
4	OXA 20 µg	/	/	7.50 ± 0.87	
5	CDDP 5 µg	/	/	7.91 ± 0.86	
6	BLM 5 µg	2.73 ± 0.28		/	/
7	OXA 85 µg	3.95 ± 0.65		/	/
8	CDDP 40 µg	3.36 ± 0.57		/	/
9	BLM 0.4 µg/ECT	/	/	13.71 ± 1.07	*1
10	BLM 0.75 µg/ECT	/	/	14.26 ± 1.57	*1
11	BLM 1.5 µg/ECT	/	/	**15.51 ± 1.87**	*1
12	BLM 2.5 µg/ECT	15.82 ± 1.03	*1	/	/
13	BLM 5 µg/ECT	16.85 ± 0.82	*1	/	/
14	BLM 7.5 µg/ECT	**18.42 ± 0.94**	*1	/	/
15	OXA 10 µg/ECT	/	/	13.51 ± 1.04	*1
16	OXA 20 µg/ECT	/	/	14.41 ± 1.47	*1
17	OXA 30 µg/ECT	/	/	**15.63 ± 1.45**	*1
18	OXA 60 µg/ECT	15.12 ± 1.67	*1	/	/
19	OXA 85 µg/ECT	**17.77 ± 1.96**	*1	/	/
20	OXA 100 µg/ECT	18.96 ± 1.41	*1	/	/
21	CDDP 2.5 µg/ECT	/	/	14.06 ± 1.2	*1
22	CDDP 5 µg/ECT	/	/	12.90 ± 1.15	*1
23	CDDP 10 µg/ECT	/	/	**19.18 ± 1.88**	*1, *22, *21
24	CDDP 30 µg/ECT	22.53 ± 1.48	*1	/	/
25	CDDP 40 µg/ECT	**22.65 ± 1.48**	*1	/	/
26	CDDP 50 µg/ECT	24.84 ± 1.01	*1	/	/

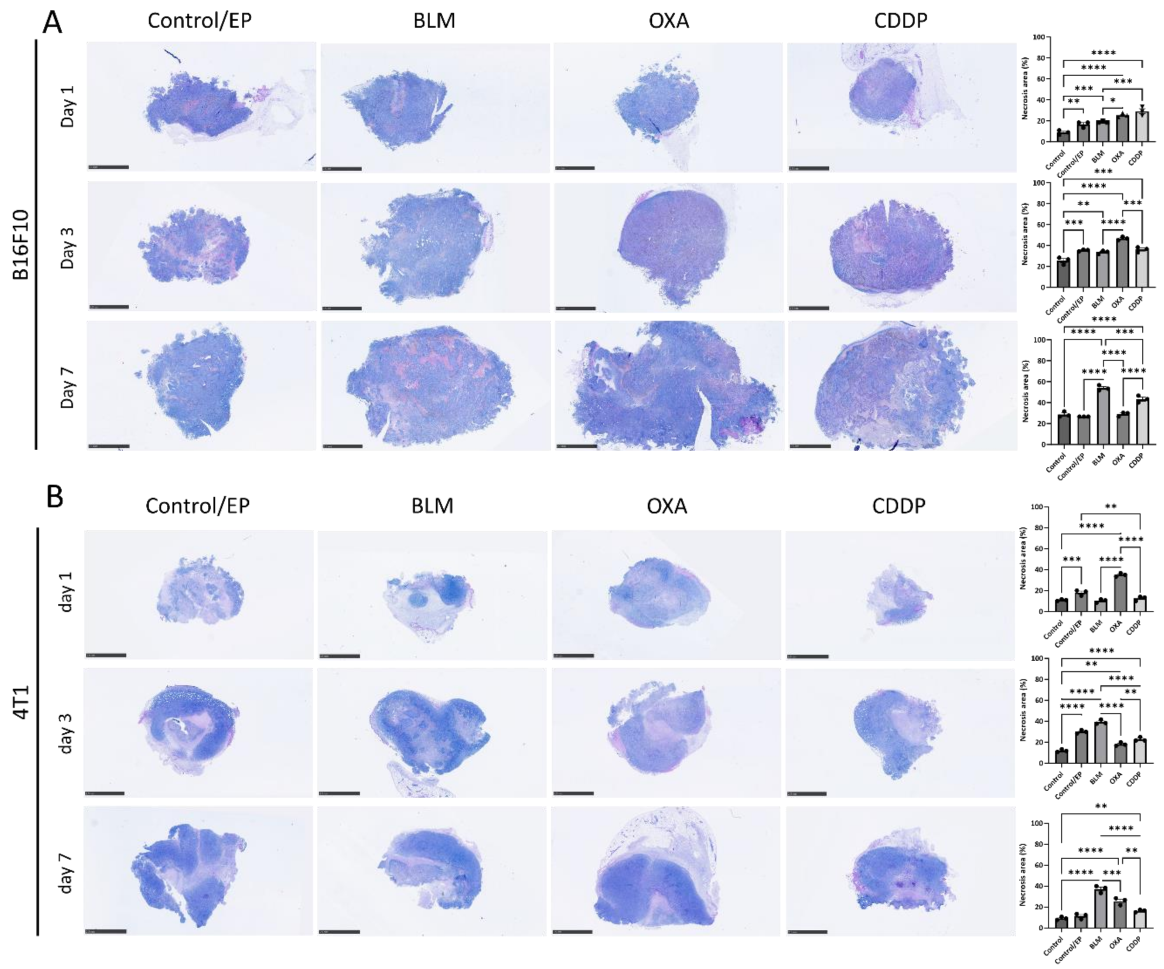

Figure S3. ECT induces necrosis. Necrotic areas in control groups one, three and seven days after the treatment in **(A)** B16F10 and **(B)** 4T1 tumors. Scale bar: 2.5 mm. BLM: bleomycin, OXA: oxaliplatin, CDDP: cisplatin. (n = 3; AM ± SE and individual measurements are presented; * $p \leq 0.05$, ** $p \leq 0.01$, *** $p \leq 0.001$, **** $p \leq 0.0001$).

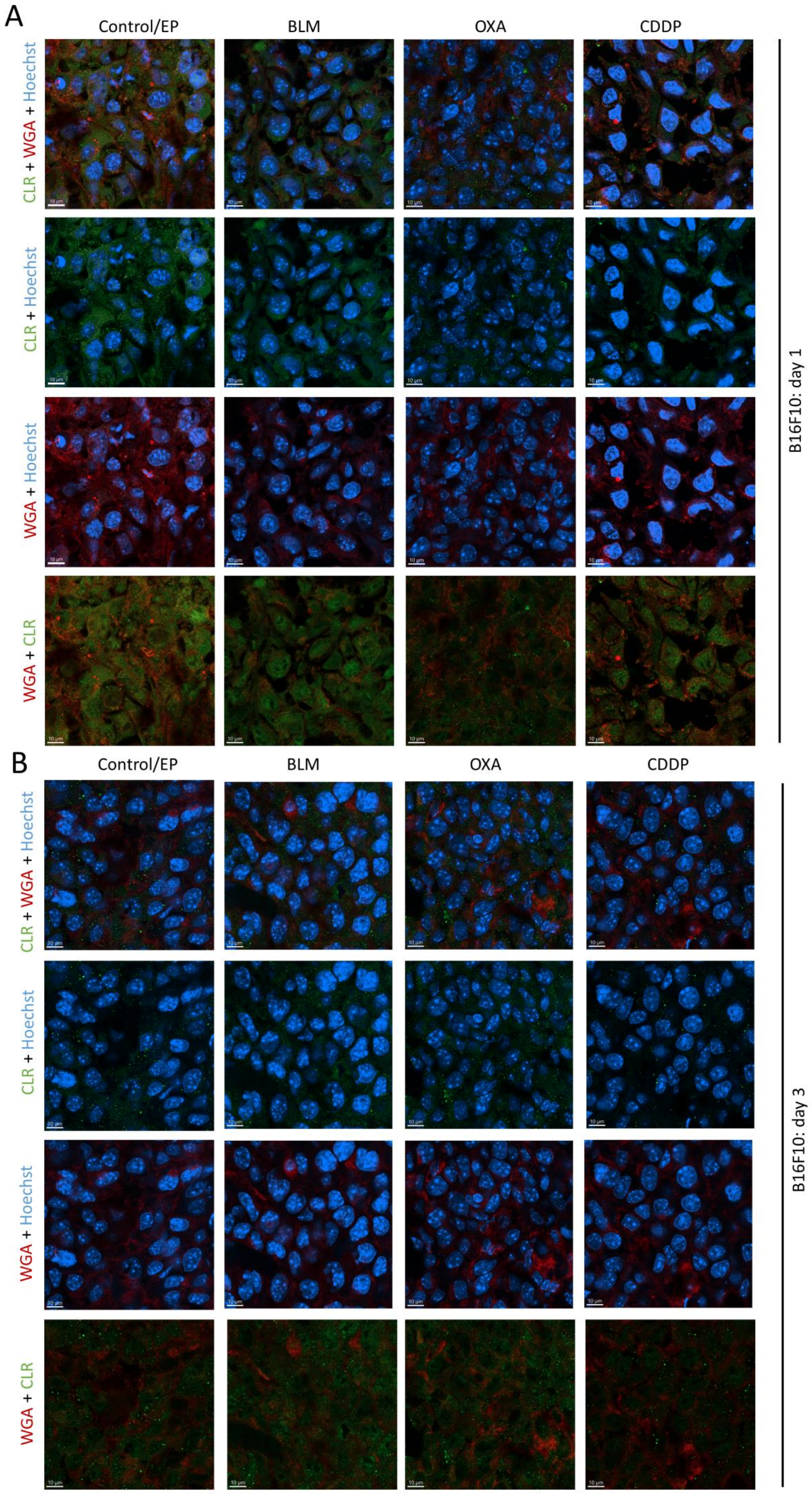

A
Control/EP
BLM
OXA
CDDP
CLR + WGA + Hoechst
CLR + Hoechst
WGA + Hoechst
WGA + CLR
B16F10: day 1
B
Control/EP
BLM
OXA
CDDP
CLR + WGA + Hoechst
CLR + Hoechst
WGA + Hoechst
WGA + CLR
B16F10: day 3

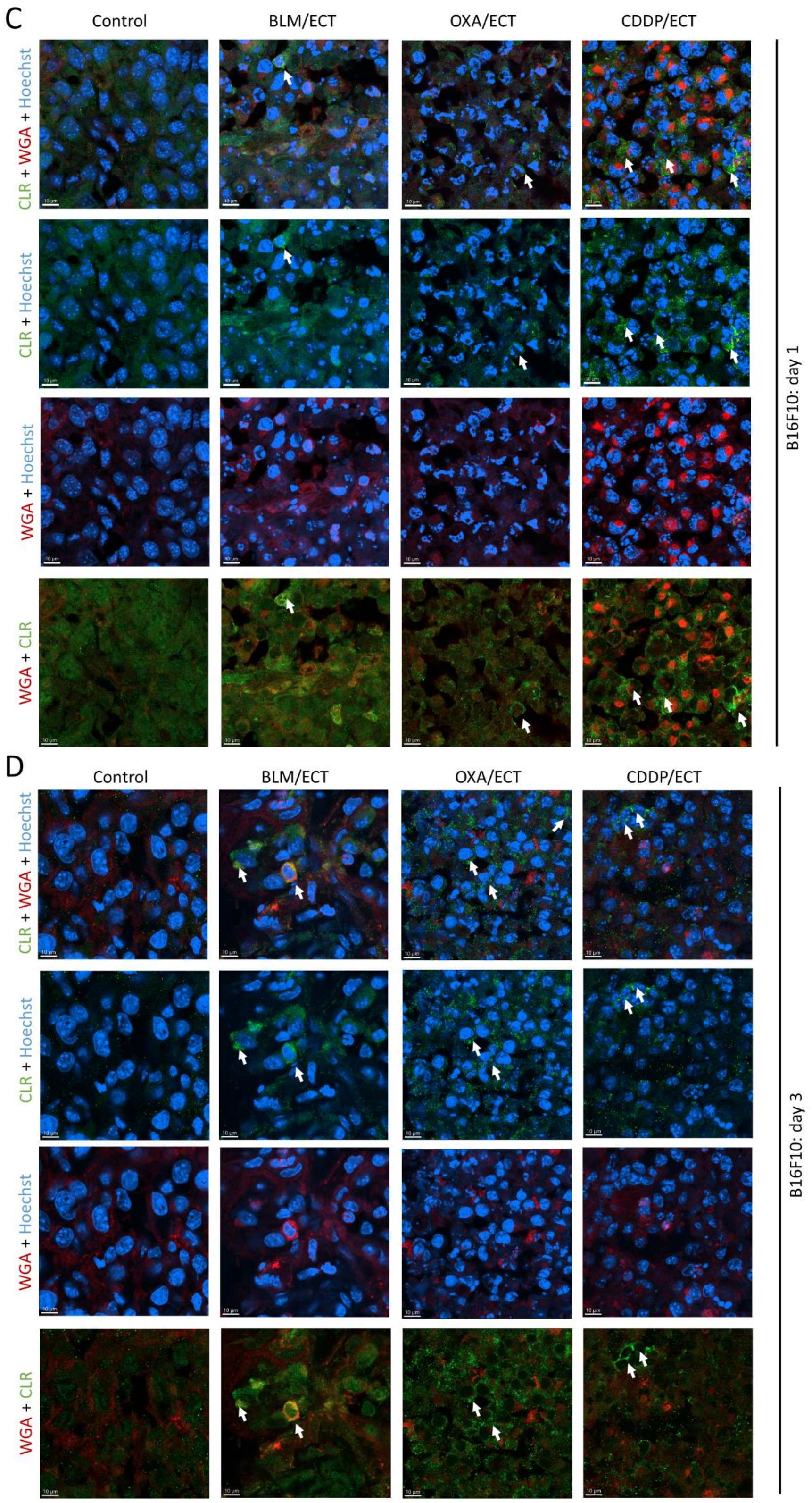

Figure S4. ECT induces translocation of calreticulin to plasma membrane of B16F10 tumors. (A) Control groups on day 1 and **(B)** day 3, as well as **(C)** ECT groups on day 1 and **(D)** on day 3 in B16F10 tumors. Arrows indicate calreticulin translocation to plasma membrane. CLR (green): calreticulin staining, Hoechst (blue): DNA (nuclei) staining, WGA (red): wheat germ agglutinin staining of membranes. Scale bar: 10 µm. BLM: bleomycin, OXA: oxaliplatin, CDDP: cisplatin.

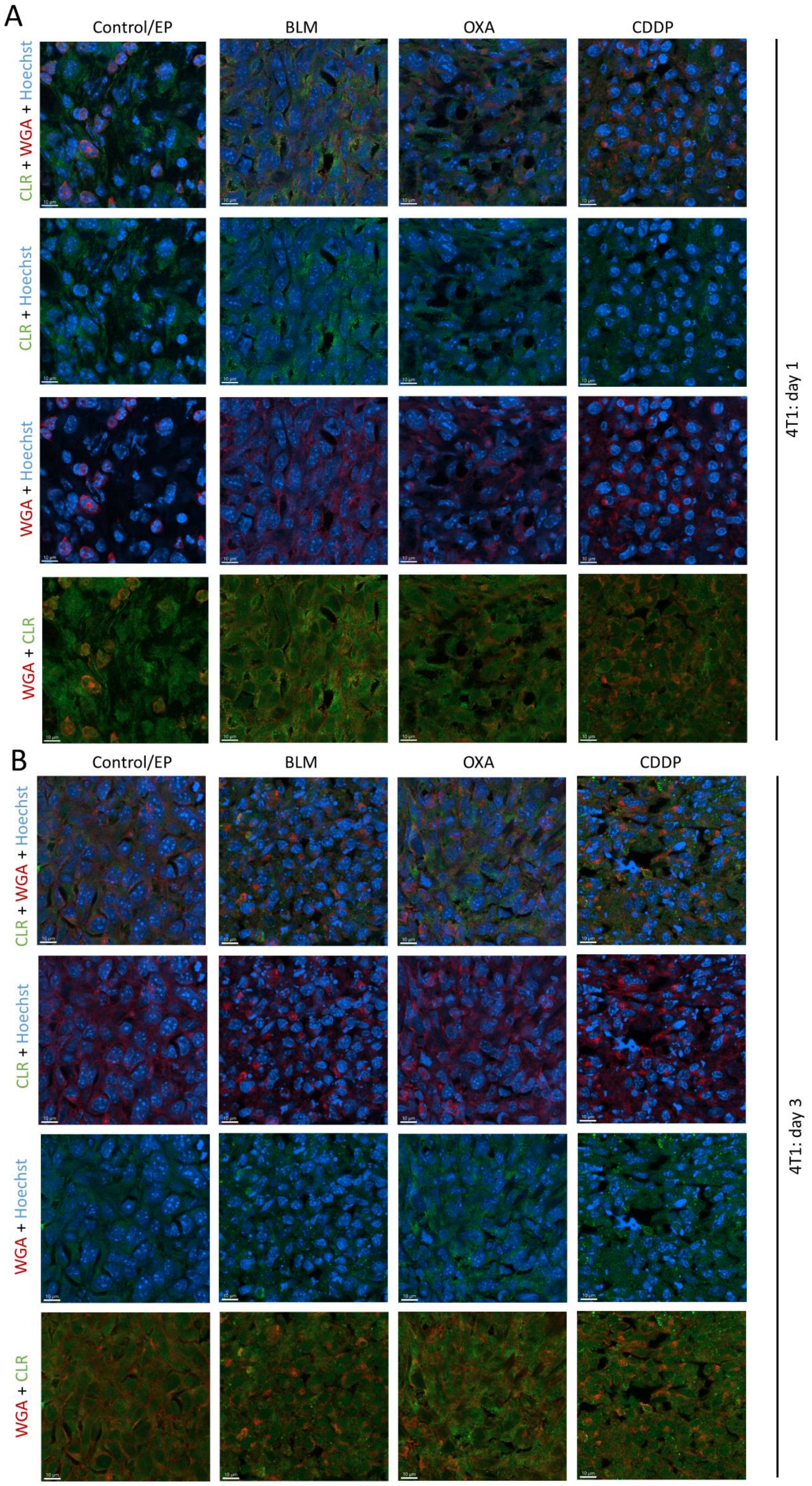
A
Control/EP
BLM
OXA
CDDP
CLR + WGA + Hoechst
CLR + Hoechst
WGA + Hoechst
WGA + CLR
4T1: day 1
B
Control/EP
BLM
OXA
CDDP
CLR + WGA + Hoechst
CLR + Hoechst
WGA + Hoechst
WGA + CLR
4T1: day 3

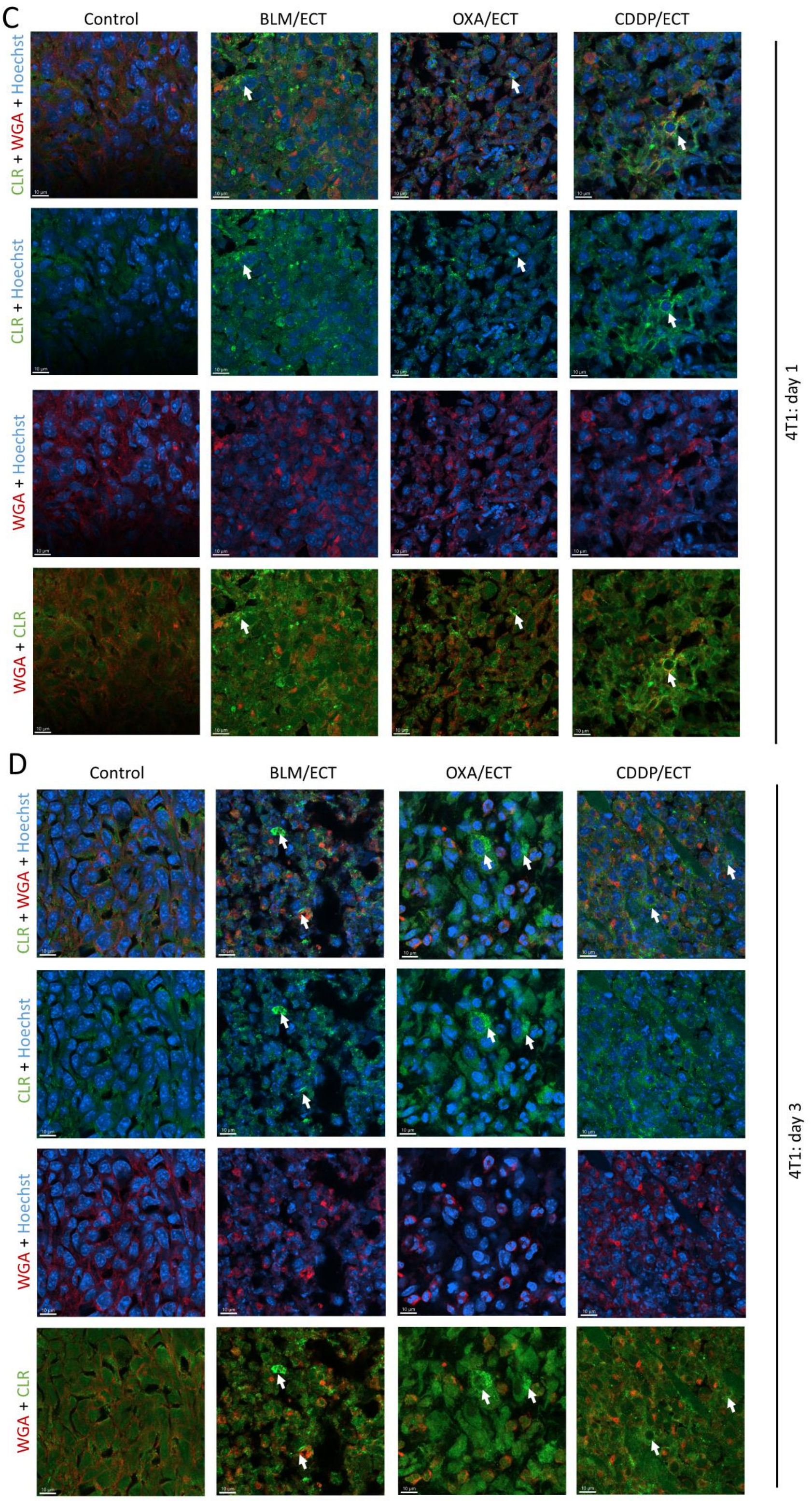

Figure S5. ECT induces calreticulin translocation to plasma membrane of 4T1 tumors. (A) Control groups on day 1 and **(B)** day 3, as well as **(C)** ECT groups on day 1 and **(D)** on day 3 in 4T1 tumors. Arrows indicate calreticulin translocation to plasma membrane. CLR (green): calreticulin staining, Hoechst (blue): DNA (nuclei) staining, WGA (red): wheat germ agglutinin staining of membranes. Scale bar: 10 μm. BLM: bleomycin, OXA: oxaliplatin, CDDP: cisplatin.

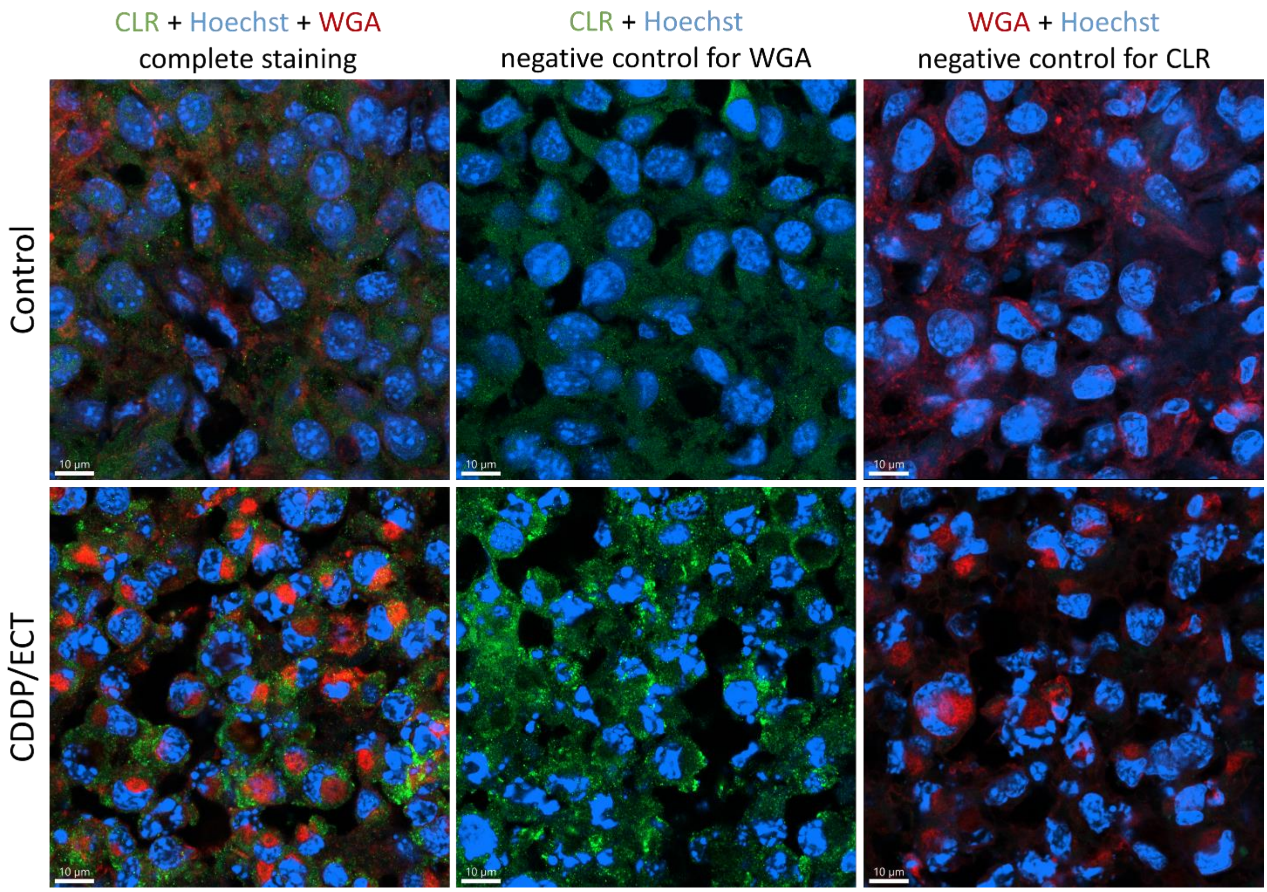

Figure S6. IFC control groups. Representative micrographies of Control B16F10 tumors and B16F10 tumors treated with ECT using CDDP are presented after complete staining (CLR + WGA + Hoechst) and stainings for CLR or WGA only. CLR (green): calreticulin staining, Hoechst (blue): DNA (nuclei) staining, WGA (red): wheat germ agglutinin staining of membranes. Scale bar: 10 µm.

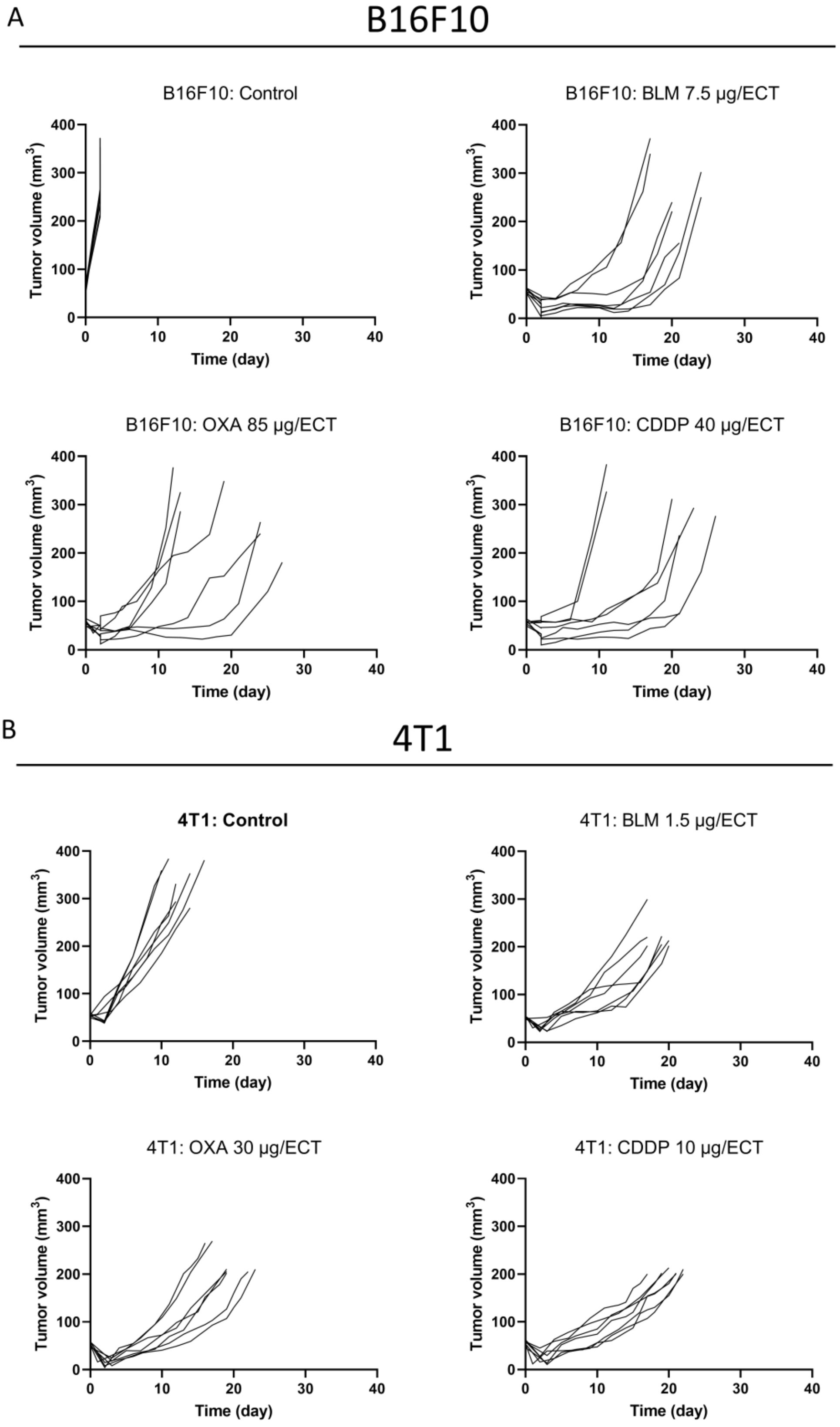

Figure S7. ECT in NUDE mice. Tumor growth curves of individual immunodeficient NUDE mice bearing **(A)** B16F10 and **(B)** 4T1 tumors are shown after ECT with BLM, OXA or CDDP (n = 6-8). BLM: bleomycin, OXA: oxaliplatin, CDDP: cisplatin.

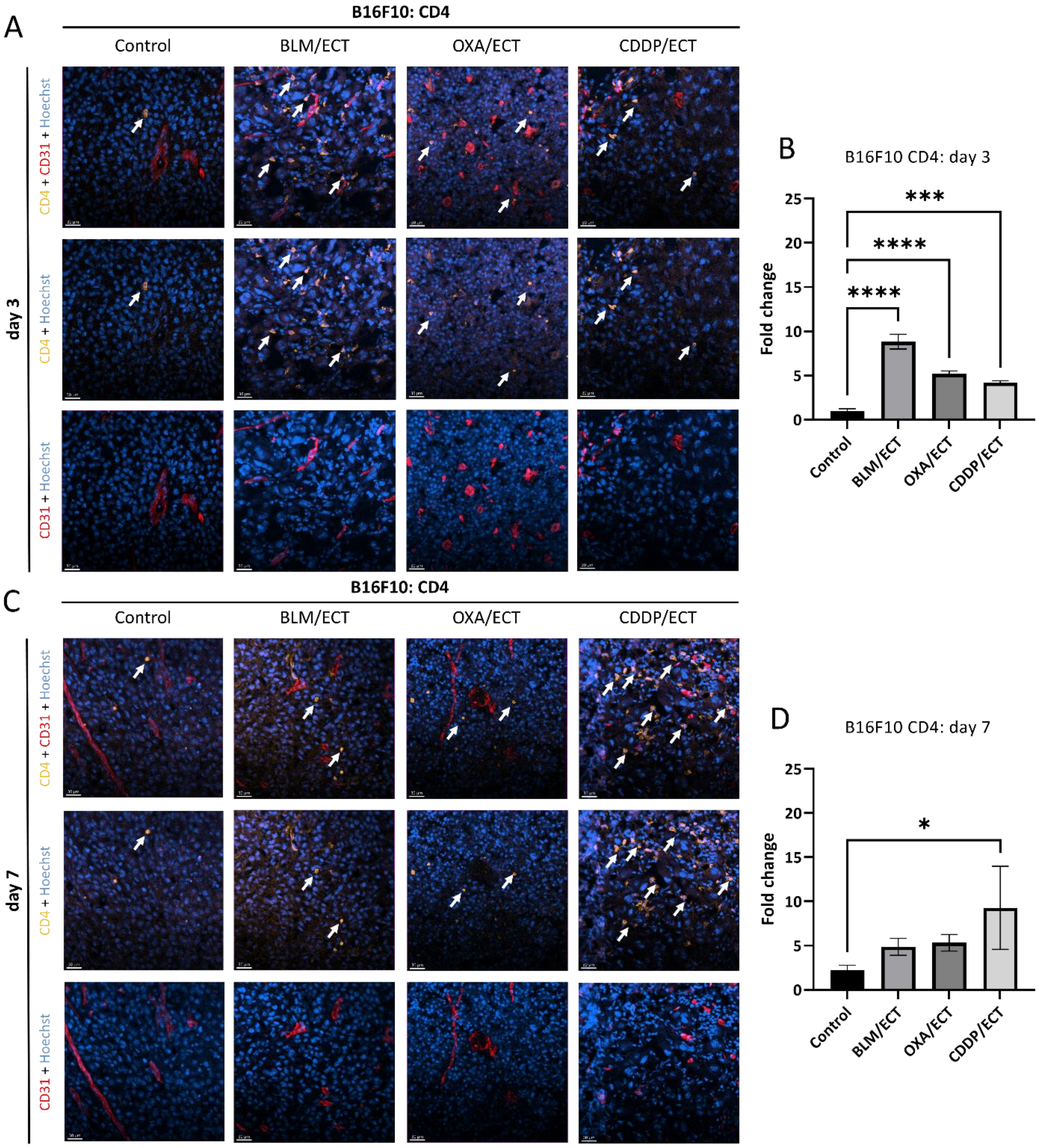

Figure S8. **ECT leads to infiltration of B16F10 tumors by CD4 positive immune cells.** Tumors were harvested three **(A, B)** and seven **(C, D)** days after the therapy. Hoechst (blue): DNA (nuclei) staining, CD4 (orange): CD4 positive immune cells, CD31 (red): vessels. Arrows indicate IFC positive cells. Scale bar: 30 µm. (n=3; AM ± SE; ns: p ≥0.05; *p 0.01 – 0.05; **p 0.001 – 0.01; ***p 0.0001 – 0.001; ****p <0.0001)

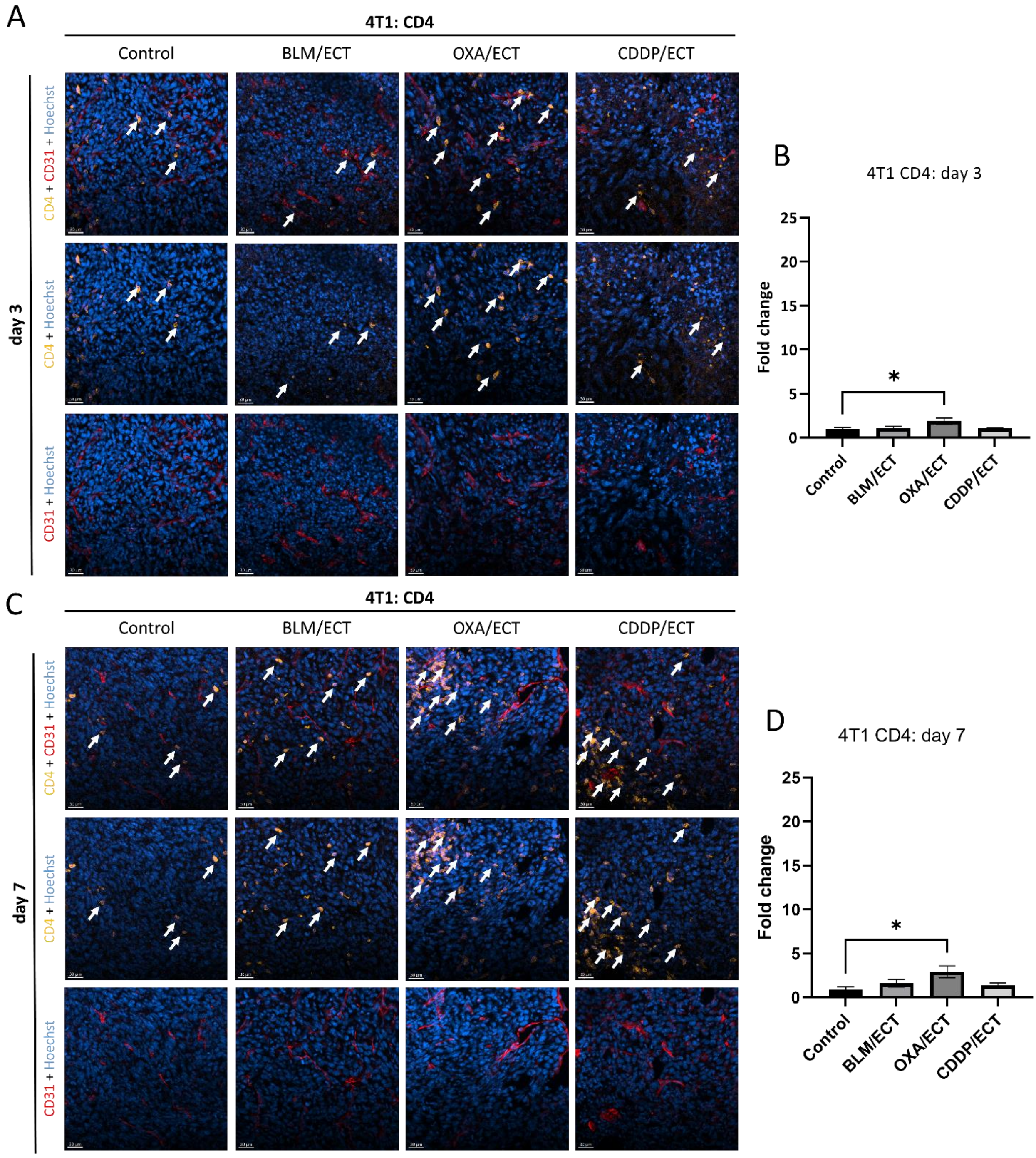

Figure S9. ECT leads to infiltration of 4T1 tumors by CD4 positive immune cells. Tumors were harvested three **(A, B)** and seven **(C, D)** days after the therapy. Hoechst (blue): DNA (nuclei) staining, CD4 (orange): CD4 positive immune cells, CD31 (red): vessels. Arrows indicate IFC positive cells. Scale bar: 30 µm. (n=3; AM ± SE; ns: p ≥0.05; *p 0.01 – 0.05; **p 0.001 – 0.01; ***p 0.0001 – 0.001; ****p <0.0001)

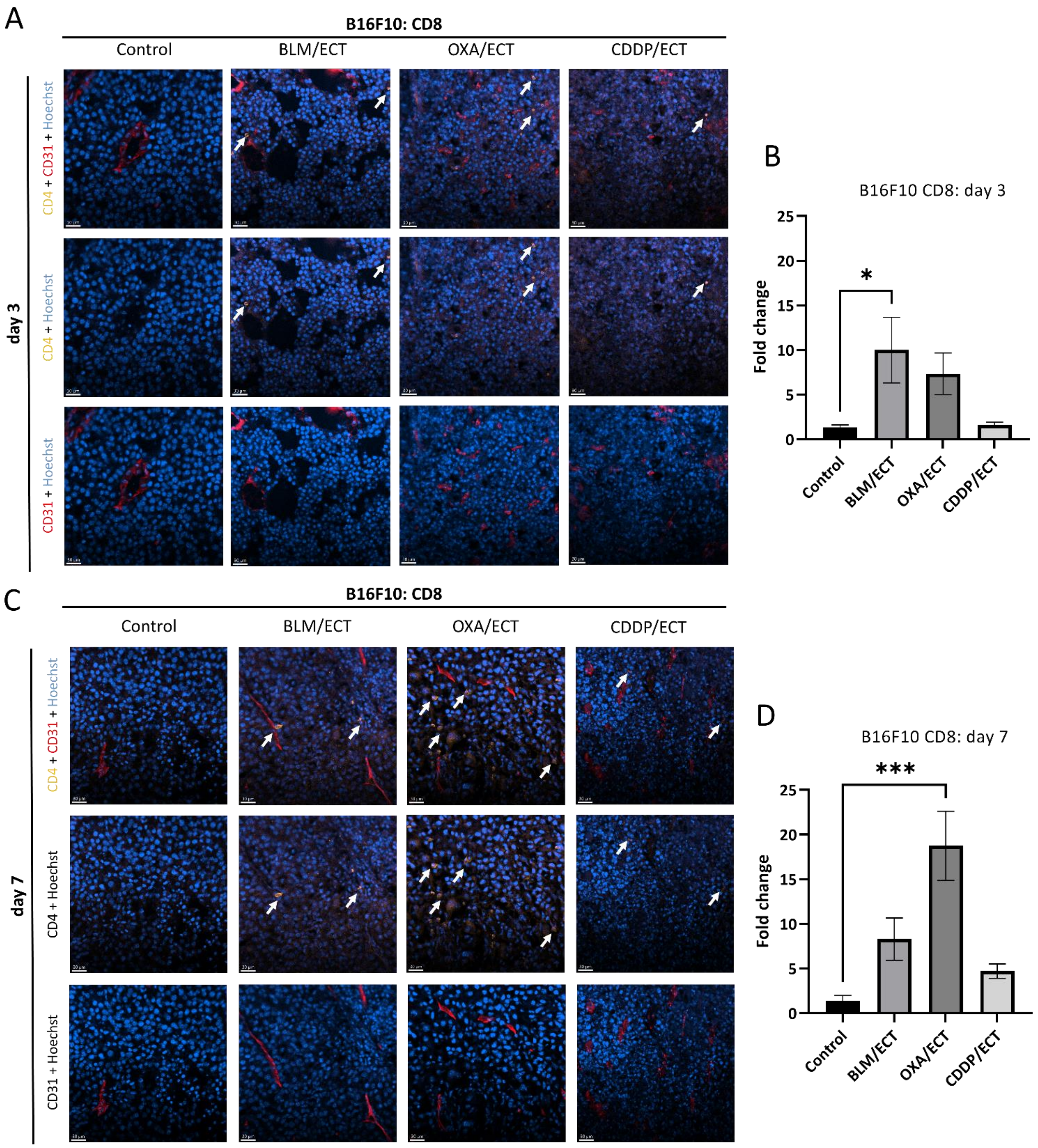

Figure S10. **ECT leads to infiltration of B16F10 tumors by CD8 positive immune cells.** Tumors were harvested three **(A, B)** and seven **(C, D)** days after the therapy. Hoechst (blue): DNA (nuclei) staining, CD8 (orange): CD8 positive immune cells, CD31 (red): vessels. Arrows indicate IFC positive cells. Scale bar: 30 μm. (n=3; AM ± SE; ns: p ≥0.05; *p 0.01 – 0.05; **p 0.001 – 0.01; ***p 0.0001 – 0.001; ****p <0.0001)

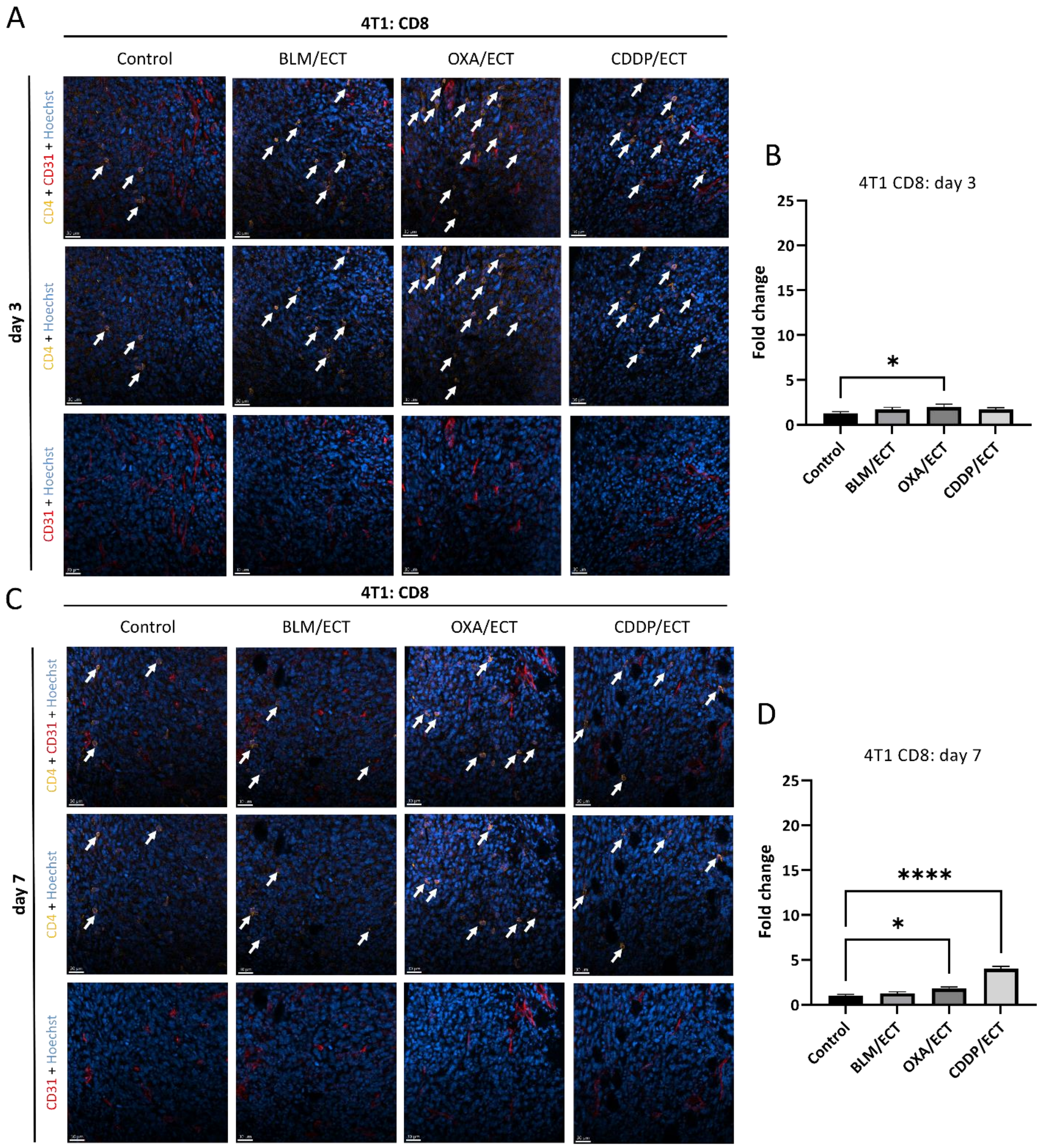

Figure S11. **ECT leads to infiltration of 4T1 tumors by CD8 positive immune cells.** Tumors were harvested three **(A, B)** and seven **(C, D)** days after the therapy. Hoechst (blue): DNA (nuclei) staining, CD8 (orange): CD8 positive immune cells, CD31 (red): vessels. Arrows indicate IFC positive cells. Scale bar: 30 µm. (n=3; AM ± SE; ns: p ≥0.05; *p 0.01 – 0.05; **p 0.001 – 0.01; ***p 0.0001 – 0.001; ****p <0.0001)

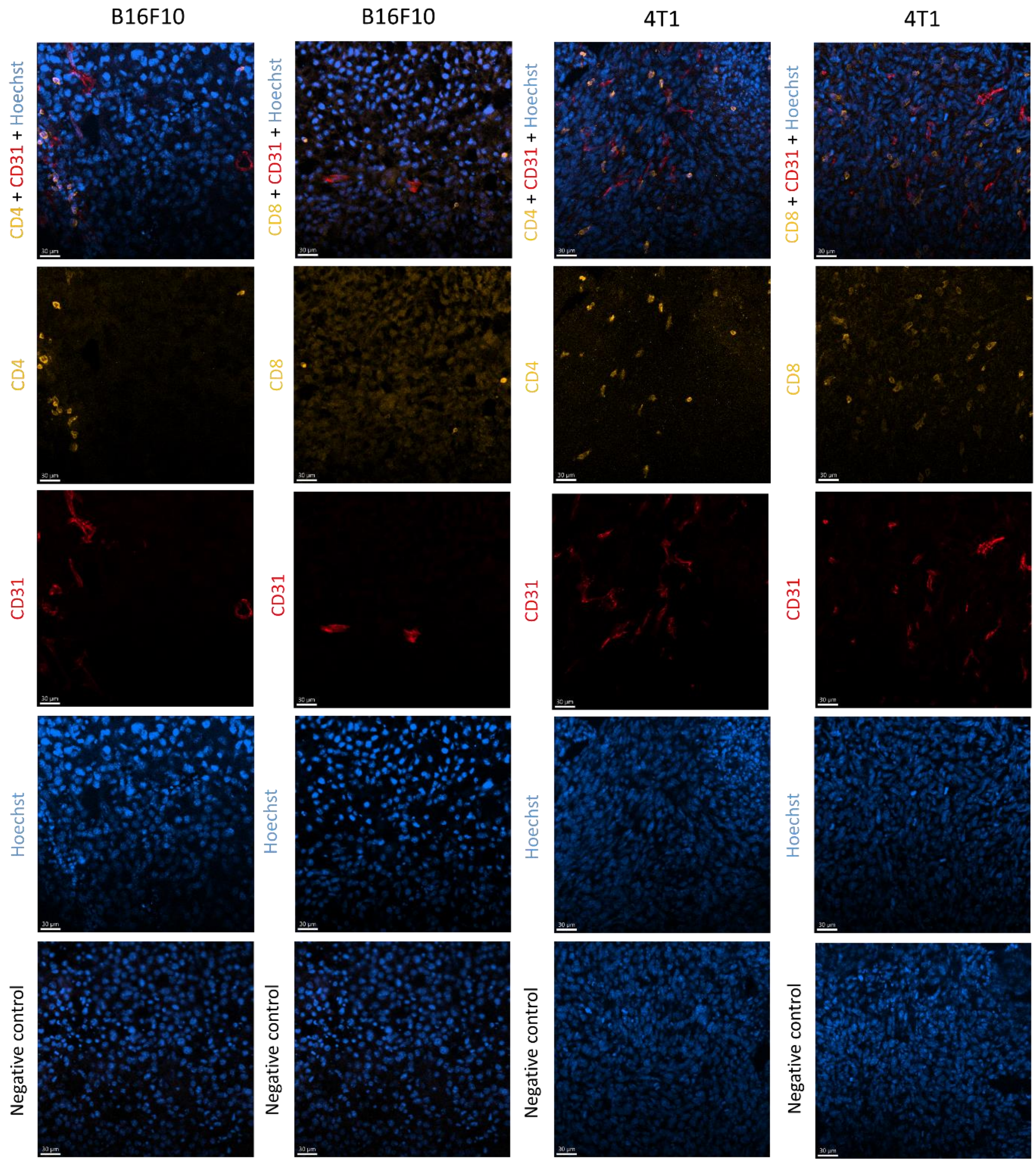

Figure S12. **IFC staining of B16F10 and 4T1 tumors**. Complete staining of tumors after ECT and respective negative controls are presented. Hoechst (blue): DNA (nuclei) staining, CD4 or CD8 (orange): CD4 or CD8 positive immune cells, CD31 (red): vessels. Scale bar: 30 µm.
